# Fate mapping RNA-sequencing reveal Malat1 regulates Sca1^+^ progenitor cells to vascular smooth muscle cells transition in vascular remodeling

**DOI:** 10.1007/s00018-023-04762-3

**Published:** 2023-04-06

**Authors:** Lingxia Lyu, Zhoubin Li, Zuoshi Wen, Yongchun He, Xuliang Wang, Liujun Jiang, Xuhao Zhou, Chengchen Huang, Yutao Wu, Ting Chen, Xiaogang Guo

**Affiliations:** 1 Department of Cardiology, The First Affiliated Hospital, College of Medicine, Zhejiang University, Hangzhou, China; 2 Alibaba-Zhejiang University Joint Research Center of Future Digital Healthcare, Hangzhou, China; 3 Kidney Disease Center, The First Affiliated Hospital, College of Medicine, Zhejiang University, Hangzhou, China

**Keywords:** Sca1^+^ cells, Arteriovenous fistula, Malat1, Single-cell sequencing, Vascular remodeling

## Abstract

Regeneration of smooth muscle cells (SMCs) is vital in vascular remodeling. Sca1^+^ stem/progenitor cells (SPCs) can generate de novo smooth muscle cells after severe vascular injury during vessel repair and regeneration. However, the underlying mechanisms have not been conclusively determined. Here, we reported that lncRNA Metastasis-associated lung adenocarcinoma transcript 1 (Malat1) was down-regulated in various vascular diseases including arteriovenous fistula, artery injury and atherosclerosis. Using genetic lineage tracing mice and veingraft mice surgery model, we found that suppression of lncRNA Malat1 promoted Sca1^+^ cells to differentiate into SMCs in vivo, resulting in excess SMC accumulation in neointima and vessel stenosis. Genetic ablation of Sca1^+^ cells attenuated venous arterialization and impaired vascular structure normalization, and thus, resulting in less Malat1 down-regulation. Single cell sequencing further revealed a fibroblast-like phenotype of Sca1^+^ SPCs-derived SMCs. Protein array sequencing and in vitro assays revealed that SMC regeneration from Sca1^+^ SPCs was regulated by Malat1 through miR125a-5p/Stat3 signaling pathway. These findings delineate the critical role of Sca1^+^ SPCs in vascular remodeling and reveal that lncRNA Malat1 is a key regulator and might serve as a novel biomarker or potential therapeutic target for vascular diseases.

**Supplementary Information:**

The online version contains supplementary material available at 10.1007/s00018-023-04762-3.

## Introduction

Veingraft is an important alternative surgical approach for many types of vascular reconstruction, for instance, arteriovenous graft (AVG) for hemodialysis patients. Currently, vein-artery anastomosis for arteriovenous fistula (AVF) is recommended [1, 2]. However, vascular access dysfunction is a major cause of morbidity and hospitalization in the hemodialysis population [3]. The most common dysfunctional etiology of both vascular modalities is venous stenosis as a result of neointimal hyperplasia [4]. By reducing the luminal diameter, this complication leads to reduced blood flow and increased risk for thrombosis [5]. Smooth muscle cell (SMC) is a key component in neointimal hyperplasia in venous stenosis [6, 7]. Apart from venous stenosis, another cause of access failure is an initial failure to mature [4], arising from inadequate smooth muscle generation to facilitate venous wall thickening [8]. Recent studies report that stem/progenitor cells (SPCs) derived from blood and vessel walls contribute to SMC accumulation [9, 10]. Roostalu et al. reported that during the early stages of vascular injury, locally existing vascular SMCs are involved in neovascular intimal hyperplasia, but when vessels are severely damaged, SPCs become the main source of neointimal SMCs [11]. Tang et al*.* documented that in severe transmural injury, stem cell antigen 1 (Sca-1) marked SPCs have the potential to differentiate into SMCs. Sca1^+^SPCs-derived SMCs constitute over 40% of total SMCs in remodeled vessels, indicating a critical and important contribution to functional recovery of vessels after injury [12]. Thus, characteristics of Sca1^+^ SPC-derived SMCs and the mechanisms involved in their differentiation should be elucidated.

Metastasis-associated lung adenocarcinoma transcript 1 (Malat1) is an abundantly expressed and exceptionally conserved nuclear lncRNA [13]. Initially, Malat1 was identified to be associated with clinical outcomes in non-small cell lung cancer patients [14]. In addition, Malat1 regulates various cell behaviors, such as migration, differentiation, proliferation, metastasis, tumor growth and chemoresistance [15–21]. Besides, Malat1 is involved in pathogenesis of various vascular diseases by regulating endothelial, smooth muscle cell and hematopoietic stem cell functions [22–24]. Cremer et al*.* found that suppressed Malat1 level augments atherosclerotic lesion formation in mice and that Malat1 inhibits Lin^−^Sca1^+^c-kit^+^ (LSK) progenitor cell proliferation [24]. Recently, through single cell sequencing, Yu et al*.* found that Malat1 was upregulated in abdominal aortic aneurysm, which contributed to SMC reduction. Thus, Malat1 might be involved in the mechanisms through which Sca1^+^ SPCs’ differentiate into SMCs. However, its roles in vascular resident Sca1^+^ SPCs have not been fully determined.

In this study, we investigated the expression levels of Malat1 in conditions that generate SMCs, including AVF, arterial injury and atherosclerosis (AS). Then, we used the lineage tracing technique by generating *Sca1-CreER*^*T2*^*; Rosa26-tdTomato* and *Sca1-CreER*^*T2*^*; iDTR/Rosa26-tdTomato* transgenic mice and performed veingraft surgery to elucidate how Malat1 impacts Sca1^+^ SPCs in vivo. Single cell RNA sequencing of veingrafts was also conducted to reveal the transcriptomic phenotype of Sca1^+^ SPC-derived SMCs. Then, we isolated and cultured Sca1^+^ SPCs in vitro to demonstrate the underlying mechanisms of Malat1 regulation. Our results provide evidence that Sca1^+^ SPC-derived SMCs in the vessel wall play vital roles in vascular remodeling and that Malat1 is a key regulator of this process.

## Materials and methods

### Single cell RNA sequencing (scRNA seq) data and data processing

The scRNA seq data for mouse femoral arteries was acquired from GSE182232 and scRNA seq data for mouse atherosclerotic plaques was from GSE117963, both of which were downloaded from the Gene Expression Omnibus (GEO) database. The GSE182232 dataset included samples from normal femoral arteries (FA), 2 week (FAI2W) and 4 week (FAI4W) injured femoral arteries, each with 10,828, 7684, and 9240 cells, respectively. The GSE117963 dataset combined AS plaque samples from mice that were fed on a cholesterol-rich diet for 14 (AS14W) or 18 (AS18W) weeks and normal aorta (CTR); each group had 3368, 2307, and 1861 cells. scRNA seq of veingrafts was performed as previously described [25]. Briefly, veingrafts were harvested and digested. After full digestion, cell pellets were suspended in PBS and stained with a LIVE/DEAD™ Fixable Near-IR Dead Cell Stain Kit (1:1000) and Hoechst 33342 (Invitrogen, H3570, 1:1000). After washing using PBS, cells were resuspended in PBS, after which single nucleated live cells (Hoechst^+^ and Dead Cell Stain^−^) were sorted into PBS with 0.04% BSA using a BD FACS ARIA II Flow Cytometer (BD Biosciences). For CD45^−^ cells, a CD45 antibody (Invitrogen, 14-0452-82, 1:2000) was additionally used. For RFP^+^ cells, FACS sorting was based on their autofluorescence. Samples were subjected to scRNA-seq. A Chromium™ Single Cell 3′ Reagent Kit v2 or v3 chemistry (10 × Genomics) was used and a standard protocol followed. The library was generated and sequenced on a Novaseq6000 PE150 platform (Illumina) with paired-end 150 bp sequencing strategy. The 10 × Chromium procedure, library generation and sequencing were performed by Oebiotech Co., Ltd (Shanghai, China).

Raw scRNA-seq data was processed using the 10 × Genomics Cell Ranger software (version 3.1.0). The matrix resulting from pre-process was analyzed using Seurat suite version 3.2.3 in R Studio version 3.6 or 4.0. Visualization of gene expressions was performed using violin plot, feature plot, dot plot, and box plot that had been generated using Seurat functions: VlnPlot, FeaturePlot, DotPlot and BoxPlot. A subset of Sca1 positive cells was selected using the Seurat function “subset” by setting the threshold of log_2_ transformed gene expression of Ly6a ≥ 1.5. Gene ontology analysis was performed using “GOplot” in R. Trajectory analysis was performed using monocle version 2.18.0. Cell-to-cell interactions were analyzed with R package CellChat.

### Patients and vascular sample collection

Studies involving human participants were reviewed and approved by the Research Ethics Committee of the First Affiliated Hospital, School of Medicine, Zhejiang University (Institutional Review Board Approval No. 2021/330). Patients/participants (or their next of kin) provided written informed consents to participate in this study. Twenty patients were enrolled in our cohort. Normal cephalic vein samples were collected from 12 patients receiving arteriovenostomy for the first time. Arteriovenous fistula (AVF) samples were obtained from 8 patients subjected to AVF ligation after kidney transplants. In addition, clinical characteristics, including demographics, comorbidities and therapeutic drugs, as well as hematological parameters of participants were collected. These patients had been hospitalized at the First Affiliated Hospital, School of Medicine, Zhejiang University between 1st June 2021 and 30th August 2021.

### Animal experiments

All animal experiments and protocols were performed in accordance with the National Institutes of Health Guidelines for the Care and Use of Laboratory Animals and were approved by the Animal Care and Use Committee of Zhejiang University. The mice strains used in this study were: Sca1-CreER^T2^ knock-in mice (C57BL/6 background) and Rosa26-tdTomato mice (B6.Cg-Gt(ROSA)26Sortm9(CAG-tdTomato)Hze/J) which had been kindly donated by Professor Zhou Bin of the Shanghai Institute of Biochemistry and Cell Biology, Chinese Academy of Sciences, University of Chinese Academy of Sciences [26]. The Rosa26-iDTR (C57BL/6 background) strain and C57BL/6 background wild type mice were purchased from the Shanghai Model Organisms Center, Inc.

The adeno-associated virus used to knockdown Malat1 in mice was purchased from Hanbio Biotechnology Co., Ltd. (Shanghai, China), including AAV-Malat1 5′-GCAGTGATGAGCATTTAATAA-3′ and AAV-NC 5′-ACTACCGTTGTTATAGGTG-3′. Mice were given a single tail vein injection of 10^11^ genome copies of AAV vectors. After injection, they were allowed one week to recover.

8–12-week male and female mice were used as the start point (0 week) for each experiment. All mice were randomly allocated to different experimental groups. Tamoxifen (Sigma, C8267, USA) dissolved in corn oil (20 mg/ml) was administered by gavage at indicated time points (at 0.1–0.15 mg/g of mouse body weight) to activate tdTomato labeling. For ablation of Sca1^+^ cells, diphtheria toxin dissolved in PBS (1.5 μg/ml) was given by intraperitoneal injection (10 μl/g of mouse body weight). Then, veingraft surgery was performed one week after, as previously described [7]. Briefly, the vena cava (about 1 cm) was harvested from donor mice, the right common carotid artery of recipient mice was mobilized and cuffs were placed at two ends of the carotid artery. The artery was turned inside out over the cuff and ligated. The vein segment was grafted between the two ends of the carotid artery. Veingrafts were postoperatively harvested at 4 weeks by cutting implanted segments from native vessels at the cuff end.

### Immunofluorescence staining

Vascular tissues were harvested, washed using PBS and fixed for 6–8 h at 4 °C in 4% paraformaldehyde. Then, tissues were dehydrated in 30% sucrose solution overnight at 4 °C, embedded in OCT and frozen at − 80 °C. Frozen tissues were sliced into 10-μm thick sections using a cryostat (Leica, CM1950). Immunofluorescence staining was performed as described [27]. The primary antibodies used in this assay were αSMA antibody (Sigma, F3777, 1:500), SM22 (Abcam, ab14106, 1:200), CNN1 (Abcam, ab46794, 1:200), SMMHC (Abcam, ab53219, 1:200), RFP antibody (Rockland, 600-401-379, 1:50) and Ki67 (Abcam, ab53219, 1:200). The Alexa Fluor-conjugated secondary antibodies used in this study were Donkey anti-Mouse IgG (Invitrogen, A21202 for Alexa Fluor 488), Donkey anti-Rabbit IgG (Invitrogen, A31572 for Alexa Fluor 555), Donkey anti-rabbit IgG (Invitrogen, A32731 for Alexa Flour 488) and Donkey anti-goat IgG (Invitrogen, A32816 for Alexa Fluor 555). Images were taken using the Nikon A1 confocal microscope. The co-staining area was quantified using the colocalization tool of Fiji. The mean gray value was calculated using the Fiji measurement tool.

### Fluorescence in situ hybridization (FISH) assay

The FISH assays for Malat1 mounts in human vascular samples were performed using the RNA FISH Probe Kit (SA-Biotin system) from Shanghai Gene Pharma Co., Ltd, as instructed by the manufacturer: Malat1 probe 5′-TGTGT + TCTCT + TGAGGGACAGTAGGTCT + TACACACAAC + TGAAAA + TAGAA + TCCAGTCCT + TTACAGAAG + TCTCGGGCT-3′. Images were taken using the Nikon A1 confocal microscope. Colored pixel intensities in individual image areas of laser spots were quantified using the Colored Pixel Counter tool of Fiji.

### Sca1^+^ stem/progenitor cell culture

The aortic arch and root from C57BL/6 mice were harvested under sterile conditions as previously described [10]. Then, the tissue was sliced into 1 mm × 1 mm pieces and placed in a T25 flask previously coated with 0.1% gelatin (Sigma, G1393, USA). The T25 flask was inverted in a 5% CO_2_, 37 °C incubator for 2–3 h until the tissue was attached to the flask. Subsequently, the flask was overturned and the complete stem cell medium was added. The medium, which comprised DMEM (American Type Culture Collection, 30-2002, USA), 10% ES cell qualified fetal bovine serum (Embriomax, ES-009-B, USA), 10 ng/ml of leukemia inhibitory factor (Merck Millipore, LIF1050, USA), 0.1 mM 2-mercaptoethanol (Sigma, M3148, USA) and 100 U/ml Penicillin–Streptomycin solution (Cienry, CR-15140, China), was changed every other day. Cells were passaged at a ratio of 1:5 when they reached 90% confluency. Then, cells were washed using PBS, detached using 0.05% trypsin–EDTA, resuspended in complete stem cell medium and seeded in 0.04% gelatin coated flasks.

### Sca1^+^ stem/progenitor cell sorting

To ensure purity, primary Sca1^+^ SPCs were sorted using the Sca1^+^ microbeads kit (Miltenyi, 130-123-124, Germany), as instructed by the manufacturer. At 90% confluency, cells were dispersed in 0.05% EDTA trypsin and washed twice using a MACS running buffer (Miltenyi, 130-091-221, Germany). Cells were incubated with an anti-Sca1 immunomagnetic antibody for 10 min and thereafter with microbeads for 15 min at 4 °C. Then, they were resuspended in the buffer and filtered with a magnetic cell separator system. Sorted Sca1^+^ SPCs were obtained and cultured in ES qualified fetal bovine serum for 20 min on a gelatin coated flask, after which a complete stem cell culture medium was added to replace the fetal bovine serum. Sca1^+^ SPCs were sorted every 5 passages.

### Smooth muscle cell differentiation

For SMC differentiation, Sca1^+^ SPCs were cultured in an SMC-differentiation medium: DMEM (American Type Culture Collection, 30-2002, USA), 10% fetal bovine serum (Gibco, 10099-141, Australia), 10 ng/ml of TGF-β1 (R&D systems, 7666-MB-005, USA), and 100 U/ml penicillin–streptomycin solution (Cienry, CR-15140, China). After transfections with siMalat1 and the negative control for 4–6 h, Opti-MEM was replaced with an SMC-differentiation medium.

### Cell transfection

Sca1^+^ SPCs were seeded in 12-well plates that had been previously coated with 0.04% gelatin and cultured to ~ 70% confluence. Before transfection, the medium was replaced with Opti-MEM (Gibco, 31985-070, USA). Cell transfections were conducted using the Lipofectamine RNAiMAX reagent (Invitrogen, 13778150, USA), as instructed by the manufacturer. Plasmids were transfected with DNA transfection reagent (Neofect, TF20121201, China). After 4–6 h of transfection, the medium was replaced with the SMC-differentiated medium or stem cell medium for different purposes. The plasmids for Malat1 overexpression were built on pcDNA3.4 vector (pCMV-MCS-WRPE-Neo). Selected sequences for knockdown and microRNA were: siMalat1, 5′-GAGCAAAGGAAGTGGCTTA-3′; siStat3, 5′-GGGUCUGGCUAGACAAUAUTT-3′; miR-125a-5p mimic, 5′-UCCCUGAGACCCUUUAACCUGUGA-3′; and miR-125a-5p inhibitor, 5′-UCACAGGUUAAAGGGUCUCAGGGA-3′.

### RNA extraction and reverse transcription

Total RNA was extracted using the EZ-press RNA Purification Kit (EZBioscience, B0004D, USA) according to the manufacturer’s instructions. RNA concentration was measured by a nanodrop spectrophotometer ND-2000 (Thermo Scientific, UK) at 280 nm and reverse transcribed using a Prime Script RT master mix (Takara, RR036A, Japan). The reaction system contained 500 ng RNA, 2 μl RT master mix and enough RNase-free water to make up 10 μl of total volume. The mixture was incubated at 37 °C for 15 min and at 85 °C for 2 min. Finally, cDNA was diluted with DEPC-treated water to a final concentration of 5 ng/μl.

### Quantitative real time polymerase chain reaction (qRT-PCR)

Quantitative real time PCR was performed using the SYBR Premix Ex Taq (TaKaRa, RR820A, Japan) on an Applied Biosystems 7500 Fast Real-time PCR system (Life Technologies, Inc.). Target genes were amplified in a duplex of 10 μl PCR mixtures (5 μl SYBR Premix Ex Taq, 1 μl cDNA template, 1 μl optimized primers and 3 μl DEPC water), loaded in 96-well or 384-well plates. Relative expressions of markers were analyzed by the 2^−ΔΔCt^ method (normalized to Tubulin). The primers used were: Malat1, F: 5′-CGAGGAAGCTCCATAACTC-3′, R: 5′-CATAGAGGATGTAGTCCGCAGCA-3′; Tubulin, F: 5′-TAGACCCCAGCGGCAACTAT-3′, R: 5′-GTTCCAGGTTCCAAGTCCACC-3′; Acta2, F: 5′-TCCTGACGCTGAAGTATCCGAT-3′, R: 5′-GGCCACACGAAGCTCGTT ATAG-3′; Tagln, F: 5′-GATATGGCAGCA GTGCAGAG-3′, R: 5′-AGTTGGCTGTCTGTGAAGTC-3′; Myh11, F: 5′-AAGCAGCCAGCATCAAGGAG-3′, R: 5′-AGCTCTGCCATGTCCTCCAC-3′; Calponin, F: 5′-GGTCCTGCCTACGGCTTGTC-3′, R: 5′-TCGCAAAG AATGATCCCGTC-3′; miR125a-5p, F: 5′-TGCCAGTCTCTAGGTCCCTG-3′, R: 5′-GCTCCCAAGAACCTCACCTG-3′.

### Western blot analysis

Total proteins were extracted using the RIPA Lysis Buffer (Beyotime, P0013B, China) containing a Protease and Phosphatase Inhibitor Cocktail (Thermo Scientific, 78440, USA) and quantified by the Enhanced BCA Protein Assay Kit (Beyotime, P0010S, China). Then, protein samples were electrophorized and transferred to PVDF membranes (Millipore, IPVH00010, USA) using the eBlot L1 Fast Wet Transfer System (GeneScript, L00686C, Singapore). Membranes with proteins were blocked using a Blocking Buffer (Beyotime, P0252, China) for 20 min and incubated overnight at 4 °C in the presence of primary antibodies. Then, membranes were washed thrice using TBST and incubated with appropriate HRP-conjugated secondary antibodies at room temperature for 1 h. The enhanced chemiluminescence kit (Millipore, WBKLS0500, USA) was used to visualize bands in the ChemiDoc XRS^+^ System (Bio-Rad, USA). Integrated density of the bands was quantified using the Fiji software. The primary antibodies used in this study were: Tubulin (CST, 2128, 1:2000), αSMA (Proteintech, 14395-1-AP, 1:2000), SM22 (Proteintech, 10493-1-AP, 1:2000), Calponin (Proteintech, 13938-1-AP, 1:1000), SMMHC (Proteintech, 21404-1-AP, 1:1000), Stat3 (CST 4904 1:1000) and P-Stat3 (CST 9145 1:1000); HRP conjugated secondary antibody (Dawen, DW-GAR007, 1:5000).

### Cell immunofluorescence staining

Cells were fixed in 4% paraformaldehyde for 10 min at room temperature, washed thrice using PBS, incubated with 0.5% Triton X-100 (Invitrogen) for 15 min, blocked using PBS containing 5% bovine serum albumin for 1 h and incubated with primary antibodies (αSMA (Proteintech 14395-1-AP 1:2000) and SM22 (Proteintech 10493-1-AP 1:2000)) at 4 °C overnight. The next day, cells were washed thrice using PBS and incubated with the Donkey anti-Rabbit IgG antibody (Invitrogen, A31572 for Alexa Fluor 555) for 2 h at room temperature. Finally, cells were DAPI-stained to visualize the nuclei. Stained cells were observed and analyzed by Nikon A1 confocal microscopy.

### Dual-luciferase reporter gene assay

The luciferase reporter was built based on the GP-miRGLO vector and the complete 3′UTR/mutant 3′ UTR (mut-3′UTR) of Malat1 and Stat3 were cloned into it. Sca1^+^ cells were co-transfected with luciferase reporter plasmids and mimic control/miR-125-5p mimic/inhibitor control/miR-125-5p inhibitor using RNAiMAX and Neofact. Luciferase and renilla activities were detected with a Dual-luciferase Reporter Assay kit (PR-E1910, Promega, USA). Relative luciferase activities were defined as the ratio of firefly luciferase activities to renilla luciferase activities with those of the control set as 1.0. Plasmid sequences were: Stat3-mmu-miR-125a-5p WT, 5′-AACCTGCCTCAGACTACAGGCCCTCAGCAAAGCTCAGGGAGTATGGTCCTTATTCTATGC-3′; Stat3-mmu-miR-125a-5p MUT, 5′-AACCTGCCTCAGACTACAGGCCCTCAGCTTTCGAGTCCCTGTATGGTCCTTATTCTATGC-3′; Malat1-mmu-miR-125a-5p WT, 5′-CGACTGGCGCATGTACGTTTGAAGGCATGAGTTGGAAACAGGGAAGATGGAAGTGTTAGGCTAGCCGGGC-3'; Malat1-mmu-miR-125a-5p MUT, 5'-CGACTGGCGCATGTACGTTTGATCCCATCTCTTCCAAAGTCCCTAGATGGAAGTGTTAGGCTAGCCGGGC-3'.

### Protein chip sequencing

Sca1^+^ SPCs were transfected with siNC or siMalat1 and cultured with TGF-β1. Proteins were extracted and subjected to chip sequencing. Protein chip sequencing was performed on the RayBio platform using a G Series Mouse Cytokine Antibody Array 4000 (GSM-CAA-4000, RayBiotech, Peachtree Corners, GA, USA), as instructed by the manufacturer. The main steps included glass slide drying, protein loading, blocking, incubation with a biotinylated antibody, incubation with Cy3 equivalent dye-streptavidin, fluorescence detection, and GSM-CAA-4000-SW data analysis.

### Statistical analysis

Statistical analyses and preparation of diagrams were performed using SPSS 20 (IBM, USA), Fiji (NIH, USA) and GraphPad Prism 7.0 (GraphPad Software, CA, USA). All measurement data are expressed as mean ± SD for more than three independent experiments. The Student’s *t* test and Chi-square test were used for comparisons of means between groups. *P* ≤ 0.05 was the threshold for statistical significance.

## Results

### Malat1 was downregulated in human arteriovenous fistula

**Fig. 1:**
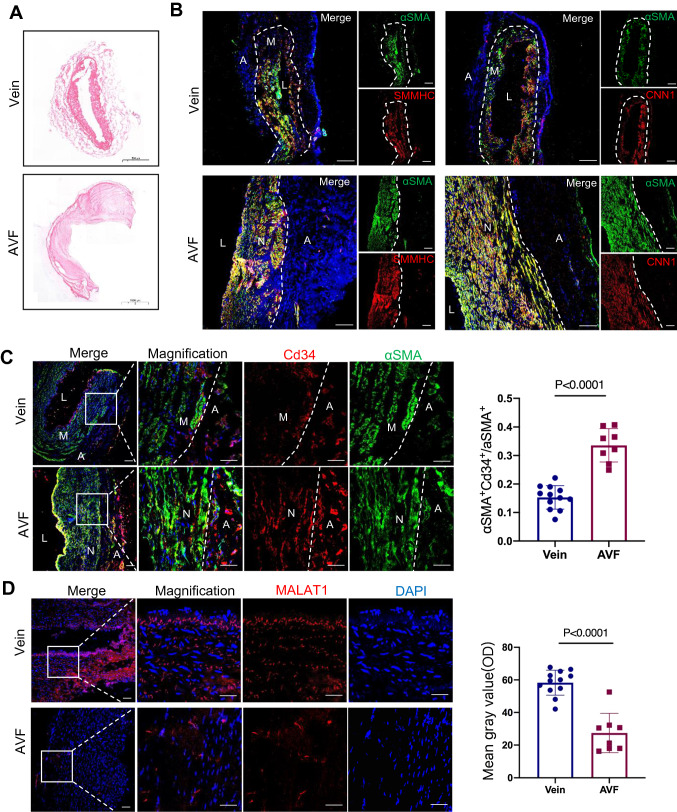
Malat1 was downregulated in human arteriovenous fistula. **A** HE staining of normal human cephalic vein (scale bar, 500 μm) and human AVF (scale bar, 1000 μm). **B** Vein and AVF immunostaining for αSMA with SMMHC and CNN1. Scale bars, 100 μm. **C** Immunostaining of vein (*n* = 12) and AVF (*n* = 8) for αSMA with Cd34, Scale bars, 100 μm. The percentage of Cd34^+^ αSMA^+^ cells among αSMA^+^ cells was quantified in the right panel using an unpaired two-tailed *t* test. *P* value was specified in the graph. **D** Fluorescence in situ hybridization (FISH) assay showing the expression of Malat1 in veins and AVFs with a magnification of the boxed region. Scale bars, 100 μm. The average optical density of Malat1 positive staining in veins and AVFs was quantified in a dot plot (right panel) using unpaired two-tailed *t* test. Data represent mean ± SD. *A* adventitia, *M* media, *L* lumen, *N* neointima

**Fig. 2:**
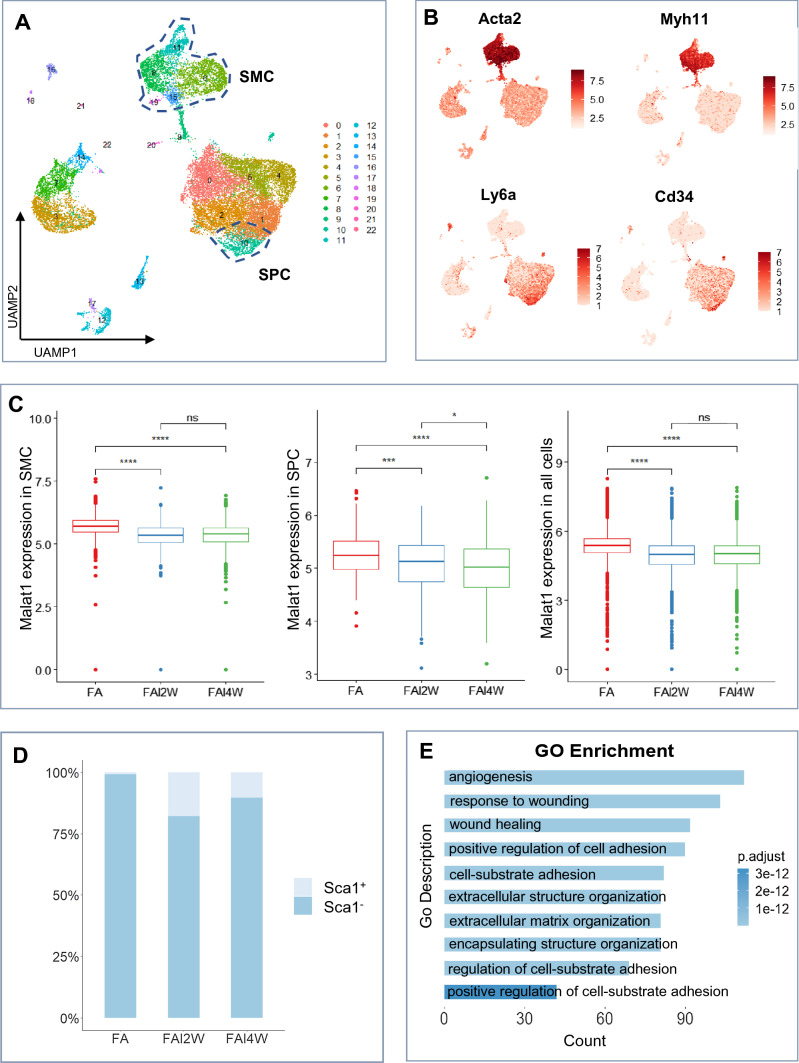
The expression of Malat1 declined in SMCs and Sca1^+^ SPCs from injured mice femoral artery. **A**, **B** Normal femoral arteries (FA) and injured femoral arteries were digested and analyzed by scRNA-seq after 2 (FAI2W) or 4 (FAI4W) weeks. Following quality control, 10,828 (FA), 7684 (FAI2W), and 9240 (FAI4W) cells were chosen for further analysis. UMAP visualization of cell types (**A**) and feature plot (**B**) of canonical cell markers (Acta2, Tagln, Ly6a, Cd34) revealed that clusters 6, 8, 11, 15, and 19 were SMCs, while cluster 10 was SPCs. **C** Box plot showing the expression levels of Malat1 in SMCs, SPCs and all cells of each group. *P* value was calculated with unpaired *t* test. **D** Bar plot showing the constitution of Sca1^+^ or Sca1^−^ cells among SMCs (clusters 6, 8, 11, 15, 19) of each group. The Sca1 expression level in Sca1^+^ cells was defined as log_2_ fold change > 1.5. **E** Differentially expressed genes between Sca1^+^ and Sca1^−^ cells were identified and analyzed using GO enrichment. The top 10 enriched biological process terms were presented. ****P < 0.0001, ***P < 0.001, ^ns^*P* ≥ 0.05

SMC regeneration plays a critical role in AVF maturation or stenosis. To investigate whether Malat1 regulates this process, we collected 12 human normal cephalic veins and 8 AVF specimens from patients. Clinical parameters and hematological characteristics for the two groups are shown in Table S1. Hematoxylin–eosin (HE) staining revealed that veins had notable arterialization and thickened vessel walls (Fig. 1A). To elucidate vessel wall composition, we assessed α-smooth muscle actin (αSMA), Calponin1(CNN1) and smooth muscle myosin heavy chain (SMMHC) expressions in veins and AVFs, which revealed that SMCs had regenerated in AVFs, compared to normal veins (Fig. 1B). Given that tunica media (M) of the vein is very thin and it’s hard to differ tunica media from neointima, we divided the vessel wall of AVF into neointima (N) and adventitia (A) to concisely and clearly clarify the results. Sca-1 is not expressed in human tissues. But Sca-1 and Cd34 are often co-expressed in mice [28], which is also observed in this study (Fig. 2B). Cells expressing Ly6a or Cd34 are overlapped. Cd34 was abundantly expressed in human AVF adventitia and neointima and massive co-staining of Cd34 and αSMA was observed, indicating the involvement of vascular progenitor cells in SMC regeneration (Fig. 1C). Then, the FISH assay was performed to assess lncRNA Malat1 levels. Of note, Malat1 was significantly downregulated in AVFs, relative to normal veins (Fig. 1D), indicating a negative correlation between SMC regeneration and Malat1 expression level.

### The expression of Malat1 declined in SMCs and Sca1^+^ SPCs during the pathological process of vascular remodeling diseases

To confirm the role of Malat1 in vascular diseases and to analyze detailed correlations of Malat1, Sca1^+^ cells and SMCs, single cell transcriptomic sequencing data of diseased vessels were downloaded from the GEO database. First, the GSE182232 dataset of injured mice femoral arteries was collected. Guidewire-induced arterial injury model mimics the pathogenesis of angioplasty-induced vessel injury [29]. During this process, SMC regeneration also contributes to neointima lesion formation [30]. This dataset includes single cell sequencing of normal femoral arteries (FA) and injured femoral arteries for 2 (FAI2W) or 4 (FAI4W) weeks. Samples of different groups were integrated for unbiased clustering analysis. Using Seurat, unsupervised clustering with UMAP analysis revealed 22 cell clusters (Fig. 2A). Feature plot (Fig. 2B) of differentially expressed genes showed that clusters 6, 8, 11, 15, 19 are SMCs, which highly express *Acta2* (encoding αSMA) and *Myh11* (encoding SMMHC). Cluster 10 highly expresses *Ly6a* (encoding Sca-1) and *Cd34* can be defined as SPCs. Notably, Malat1 levels were suppressed in injured samples, relative to normal femoral arteries. In subsets of SMCs and Sca1^+^ SPCs, Malat1 levels were also downregulated (Fig. 2C). Feature plot (Fig. 2B) revealed that some SMCs also express *Ly6a*. These cells might be Sca1^+^ SPC-derived. The bar plot (Fig. 2D) showed that the percentage of Sca1^+^ SMCs was larger in FAI2W and FAI4W, while in normal arteries, only a few SMCs expressed Sca1. Then, differentially expressed genes between Sca1^+^ SMCs and Sca1^−^ SMCs were subjected to GO (gene ontology) analysis (Fig. 2E). Biological terms including “angiogenesis”, “wound healing”, and “cell adhesion” were enriched, indicating that Sca1^+^ SMCs are involved in a responsive state and vessel repair.

Using single cell RNA sequencing data (GSE117963) from atherosclerosis mice models, we assessed Malat1 levels in Sca1^+^ SPCs and SMCs in atherosclerosis (Fig. S1), in which SMCs greatly regenerate to form lesions and play indispensable roles throughout its pathological process [31]. This dataset includes 7536 cells of AS plaques from mice fed on a cholesterol-rich diet for 14 (AS14W) or 18 (AS18W) weeks and cells of the normal aorta as control (CTR). Umap visualization (Fig. S1A) revealed basic clustering while dot plot (Fig. S1B) showed the top differentially expressed genes. SMCs were defined by *Acta2* and *Tagln* (encoding SM22), while clusters 3 and 11 were defined as SPCs as they exclusively expressed *Ly6a* and *Cd34* (Fig. S1C). Notably, compared to healthy vessels, Malat1 was downregulated in both AS groups (Fig. S1D, E), consistent with findings from human AS plaques [24]. Malat1 levels were significantly suppressed in Sca1^+^ SPCs and SMCs after AS, indicating that it might also be involved in regulation of these two cell groups during AS (Fig. S1E). These findings indicate that Malat1 suppression in Sca1^+^ SPCs and SMCs has a positive correlation with cell activation and might promote Sca1^+^ SPCs to differentiate into SMCs.

### Malat1 deficiency promotes Sca1^+^ SPCs to differentiate into SMCs in mice veingraft

**Fig. 3:**
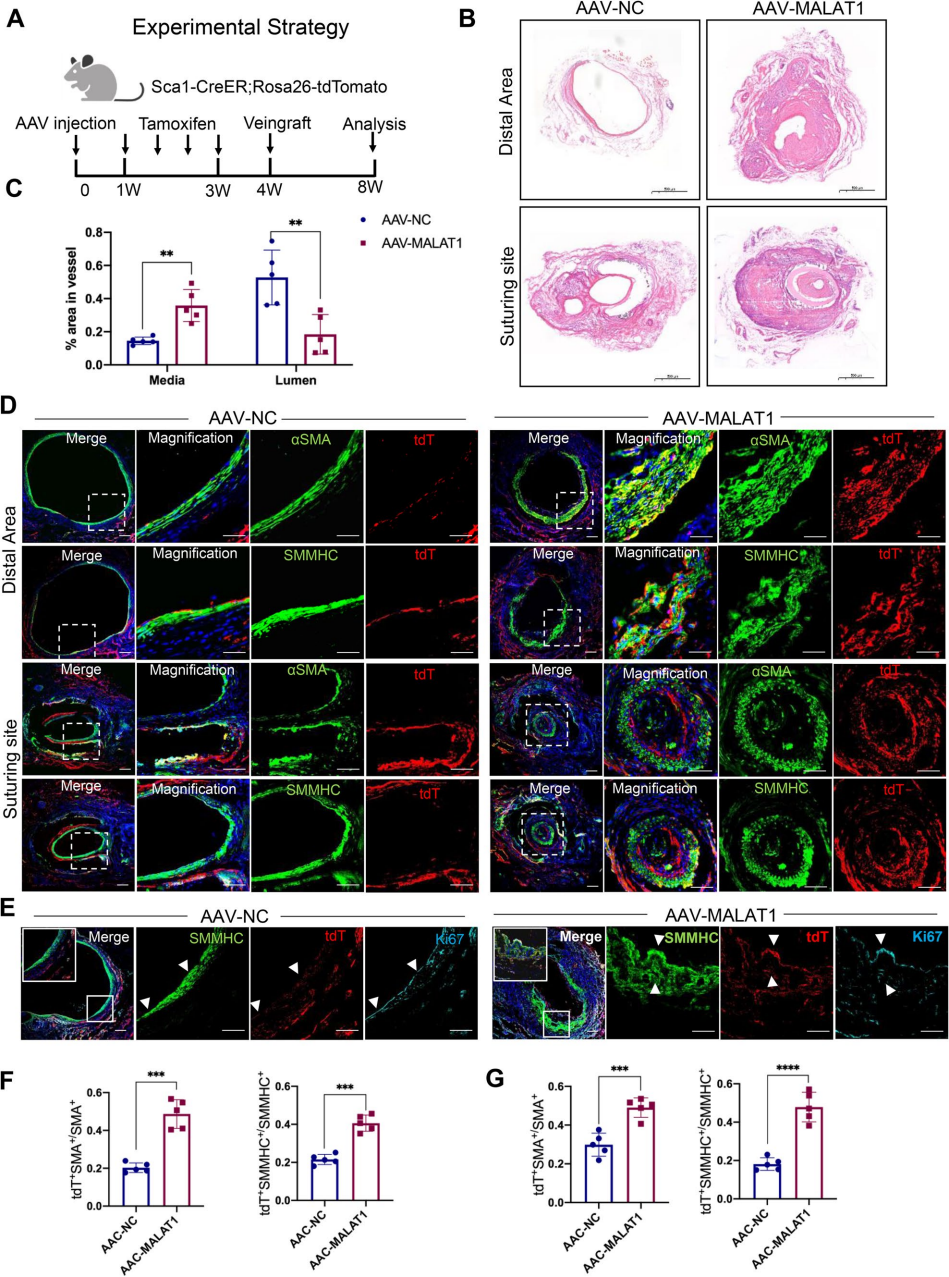
Malat1 deficiency promotes Sca1^+^ SPCs to differentiate into SMCs in mice veingraft. **A** Schematic representation of the pipeline of AAV-Malat1 or AAV-NC injection via tail vein and tamoxifen administration, followed by vein graft. The graft tissue was harvested and analyzed 4 weeks after surgery. **B** HE staining of vein grafts from AAV-NC and AAV-Malat1. Scale bars, 500 μm. **C** Average percentage of media and lumen area of total area in each group (*n* = 5) were quantified using unpaired two-tailed *t* test. Data represent mean ± SD. **D** Fluorescence staining of distal area and suturing sites of veingraft for αSMA and SMMHC with tdT (Sca1) in AAV-NC and AAV-Malat1 groups. Scale bars, 100 μm. **E** Fluorescence staining of distal area of veingraft from both groups for SMMHC, tdT and Ki67. Scale bars, 100 μm. **F** Quantification of tdT^+^SMA^+^ cells among SMA^+^ cells and tdT^+^SMMHC^+^ cells among SMMHC^+^ cells in distal area from AAV-NC and AAV-Malat1 groups. **G** Quantification of tdT^+^SMA^+^ cells among SMA^+^ cells and tdT^+^SMMHC^+^ cells among SMMHC^+^ cells in suturing sites from AAV-NC and AAV-Malat1 groups. ****P < 0.0001, ***P < 0.001, **P < 0.01

Next, we investigated whether Malat1 participates in vascular remodeling by regulating the transition of Sca1^+^ SPCs to SMCs. Here, a veingraft mouse model was used by transplanting the vena cava of a wild-type mouse onto the carotid artery of *Sca1-CreER*^*T2*^*; Rosa26-tdTomato* mice. Figure 3A depicts the entire workflow. Sca1^+^ cells are labeled by red fluorescence protein named tdTomato after being induced by tamoxifen in the transgenic mice. Following that, veingraft surgery was performed and the grafts were analyzed 4 weeks later. Fig. S2A and S2B show the longitudinal structure of veingraft model and cross-section of the distal area, where the hierarchy structure is clearer. Similar to human AVF, the vena cava undergoes an arterialization process. A large number of cytokines were released as a result of the highly elevated blood pressure and induced aggressive SMC migration and regeneration [7]. Similarly, excessive neointima hyperplasia can cause stenosis or obstruction in the veingraft, particularly in the anastomotic area. In this study, we used an adeno-associated virus to knock down Malat1(AAV-Malat1) in mice before surgery, the empty virus vector was used as control (AAV-NC). We chose to harvest the graft at the 4th week because the vessel had undergone significant remodeling and the knockdown efficacy of Malat1 was still promising, as confirmed by FISH assay. RT-PCR of Malat1 at the second and 4th week also proved that the silencing effect lasted throughout the procedure (Fig. S2C, S2D). Normal vena cava before vessel remodeling (Fig. S2E) and veingrafts from AAV-NC and AAV-Malat1 groups were all collected for analysis. As expected, HE staining showed that vena cava underwent arterialization, resulting in a thicker vessel wall in veingraft. Besides, in comparison to the AAV-NC group, neointima hyperplasia in the veingrafts was more severe in the AAV-Malat1 group, giving rise to thicker vessel walls and reduced lumen area (Fig. 3B, C). Immunofluorescence staining of normal vena cava showed that before tamoxifen treatment, almost no tdTomato signal was detected (Fig. S2F), while after tamoxifen inducing, some cells were labeled but little co-staining of SMC markers and tdTomato was observed in the smooth muscle layer (Fig. S2G). However, in the distal area of veingraft, we observed co-staining of tdTomato with αSMA, SMMHC (Fig. 3D), CNN1and SM22 (Fig. S2H) in the neointima, demonstrating that Sca1^+^ SPCs repopulate SMCs in mouse veingraft. Staining of veingraft suture sites displayed excessive SMC regeneration led to vessel obstruction (Fig. 3D). Furthermore, co-staining of tdTomato with SMC markers was remarkedly increased in AAV-Malat1 group, indicating that the lower the Malat1 expression, the more Sca1^+^ SPCs develop into SMCs (Fig. 3F, G). We also stained Ki67 of the veingraft, demonstrating that Sca1^+^ SMCs are indeed the newly proliferated cells from Sca1^+^ SPCs that repopulate the veingraft (Fig. 3E).

### Sca1^+^ cells deletion decreased SMC regeneration and restored Malat1 expression

**Fig. 4:**
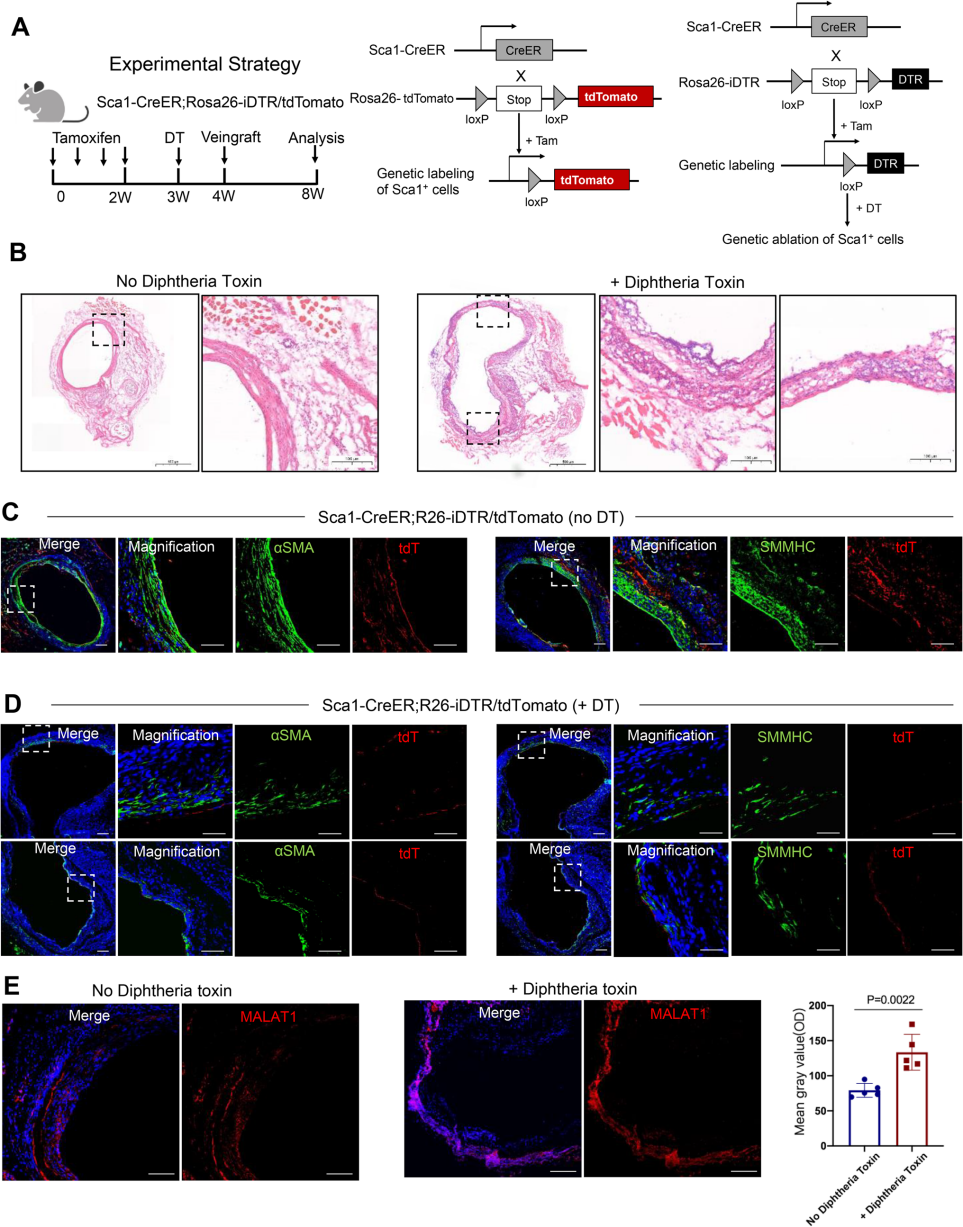
Sca1^+^ cells deletion decreased SMC generation and restored Malat1 expression. **A** Schematic representation of the pipeline of tamoxifen and diphtheria toxin (DT) administration, followed by vein graft. The graft tissue was harvested and analyzed 4 weeks after surgery. The strategy for genetic lineage tracing and ablation of Sca1^+^ cells was shown in the middle and right panels. **B** HE staining of vein grafts from Sca1-CreER^T2^; Rosa26-iDTR/tdTomato mice with or without DT treatment. Scale bars, 500 μm. For magnification of boxed areas, Scale bars, 100 μm. **C** Fluorescence staining of distal area of veingraft without DT treatment for αSMA and SMMHC with tdT. Scale bars, 100 μm. **D** Fluorescence staining of distal area of veingraft with DT treatment for αSMA and SMMHC with tdT. Scale bars, 100 μm. **E** FISH assay showing the expression of Malat1 in veingraft with or without DT treatment. Scale bars, 100 μm. The average optical density of Malat1 positive staining in both groups was quantified in the right panel using unpaired two-tailed *t* test. Data represent mean ± SD. *P* value was specified in the graph

To further address the function of Sca1^+^ cells, we crossed the S*ca1-CreER*^*T2*^*; Rosa26-tdTomato* to *Rosa26-iDTR mouse lines* [32] to allow selective ablation of Sca1^+^ cells. In the transgenic mice, tamoxifen could activate tdTomato labeling and diphtheria toxin receptor (DTR) expression on Sca1^+^ cells. Then, addition of diphtheria toxin (DT) leads to its binding with DTR, resulting in termination of protein synthesis and, thus, apoptotic death of Sca1^+^ cells [33]. Figure 4A depicted the entire workflow as well as genetic labeling and ablation strategies. We treated mice with tamoxifen and DT before performing veingraft surgery, with each procedure separated by one-week interval, and then analyzed tissues at 4 weeks of vascular repair. As a control for DT-mediated cell ablation, we used *Sca1-CreER*^*T2*^; *Rosa26-iDTR/tdTomato* mice treated with PBS instead of DT. HE staining showed that in the control group, the neointima layer was clear and dense, while in DT treated group, the hierarchy of vessel wall was disordered. The arterialization of the venous wall was inadequate, giving rise to vessel dilation and aneurysm-like structure in response to increased blood flow (Fig. 4B). Staining of SMC markers showed that in the control group, SMCs were concentric and orthogonally aligned and the smooth muscle layer was clear and thickened. But in DT treated group, SMCs were scattered throughout the vessel wall, and the smooth muscle layer was indistinct and loose. tdTomato signals were also significantly weakened. These data suggest that Sca1^+^ cells ablation resulted in inadequate smooth muscle generation to facilitate venous wall thickening. Of note, we found that in DT-treated group, Malat1 expression was higher than the control group. As discussed above, Malat1 expression in SMCs and Sca1^+^ SPCs was suppressed during vascular remodeling diseases including AVF, AS and artery injury. But in DT-treated group, because Sca1^+^ cells were ablated, SMC regeneration and related signal pathways were not fully activated, thus, resulting in inadequate Malat1 suppression compared to the control group.

### ScRNA seq reveals the transcriptomic phenotype of Sca1^+^ SPCs derived SMCs

**Fig. 5:**
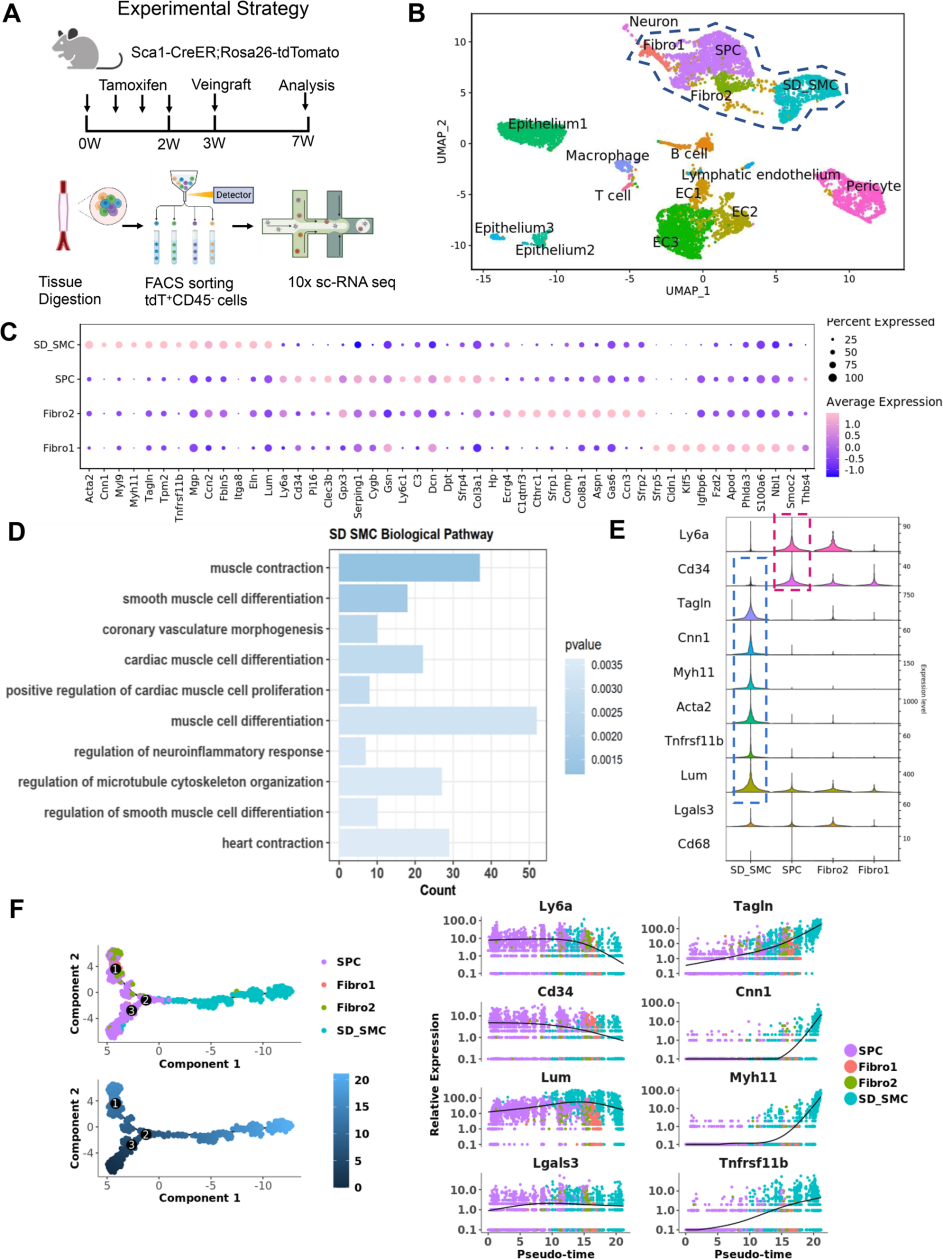
ScRNA seq reveals the transcriptomic phenotype of Sca1^+^ SPCs derived SMCs. **A** Schematic diagram showing the experimental strategy of animal experiments and single cell sequencing. **B** Umap distribution of cell types in tdTomato^+^ CD45^−^ cells isolated from vein graft tissue. **C** Canonical and top expressed genes of each cluster presented in a dot plot. **D** GO biological pathway analysis of the top differentially expressed genes in SD_SMCs from other cell types. The top 10 enriched pathways are presented. **E** A violin plot of the canonical cell markers of stem/progenitor cells (Ly6a, Cd34), SMCs (Tagln, Cnn1, Myh11 and Acta2), macrophage-like SMCs (Lgals3, Cd68), and highly-expressed markers of SD_SMCs (Tnfrsf11b, Lum) is presented. **F** Trajectory analysis of the differentiation process from SPCs to SD_SMCs; the color change from dark to light indicates the cell’s progression from an earlier to a more developed state (left panel). The expression levels of canonical cell markers are shown alongside the differentiation trajectory

Since Sca1^+^ SPCs give rise to a large number of SMCs, it is important to investigate the characteristics of these de novo SMCs. We performed scRNA sequencing on the grafted vessels. Figure 5A depicts the data generation procedure. To ensure that only Sca1^+^ SPCs derived SMCs (SD_SMC) were included in the single-cell suspension, we sorted out tdTomato^+^ cells using FACS. In the meantime, we excluded Cd45^+^ cells because it has been reported that bone marrow Sca1^+^ cells can generate inflammatory cells, the majority of which express the Cd45 marker [34], while this is not the objective of this study. First, we performed Umap clustering of all the data, including 9155 cells after quality control (Fig. 5B). Then, we selected cell clusters closely distributed to Sca1^+^ SPCs and SD_SMCs in Umap space for further analysis. Top expressed genes and canonical genes of selected clusters were presented (Fig. 5C). Then, we sorted the top differentially expressed genes in SD_SMC and performed a Gene Ontology analysis (Fig. 5D). Here, we presented the top 10 enriched biological pathway terms including “muscle cell differentiation”, “muscle contraction”, “microtubule cytoskeleton organization” etc*.*, which are typical smooth muscle related-functions. In recent decades, vascular smooth muscle cell phenotype transition under pathological and physiological conditions has been extensively and exhaustively investigated. Researchers have identified several modulated SMC phenotypes such as macrophage-like SMCs (express *Lgals3, Cd68*) [35], and most recently, fibroblast-like SMCs (express *Tnfrsf11b, Lum*) [36]. SD_SMCs did not appear to be transforming into macrophage-like SMCs because they barely express *Lgals3* and *Cd68* (Fig. 5E). In contrast, SD_SMCs exhibited a high level of *Tnfrsf11b, Lum,* and canonical SMC marker expression. In addition, we performed a Monocle analysis of the trajectory. Figure 5B shows that SPCs are in an earlier developmental status (dark blue) than SD_SMCs (light blue). We observed changes in the expression levels of representative markers of SPCs (*Ly6a, Cd34*), SMCs (*Cnn1, Tagln, Myh11*), fibroblast-like SMCs (*Tnfrsf11b, Lum*) and macrophage-like SMCs (*Lgals3, Cd68*) along the differentiation trajectory. In this pseudo-time process, *Ly6a, *and* Cd34* demonstrated a gradient of decreasing expression from SPCs (purple) to SD_SMCs (blue), whereas *Tagln, Cnn1, Myh11 *and* Tnfrsf11b* showed a significant increase in expression level, suggesting SD_SMCs is similar to fibroblast-like SMCs. To our surprise, we discovered that knocking down Malat1 could up-regulate the protein OPG in cultured Sca1^+^ cells, which is encoded by *Tnfrsf11b* (Fig. S4A). Protein chip array sequencing of isolated Sca1^+^ cells transfected with siRNA-Malat1 or siRNA normal control revealed that, among the 132 differentially expressed proteins, OPG was the most significantly up-regulated. *Tnfrsf11b* was almost exclusively expressed in SD_SMCs, as shown in the feature plot (Fig. S4B). These results also suggested that Malat1 may be related to the phenotype of SD_SMCs.

### ScRNA seq maps SD_SMCs back to whole-cell analysis and elucidate that Malat1 loss alters cell-to-cell interactions

**Fig. 6:**
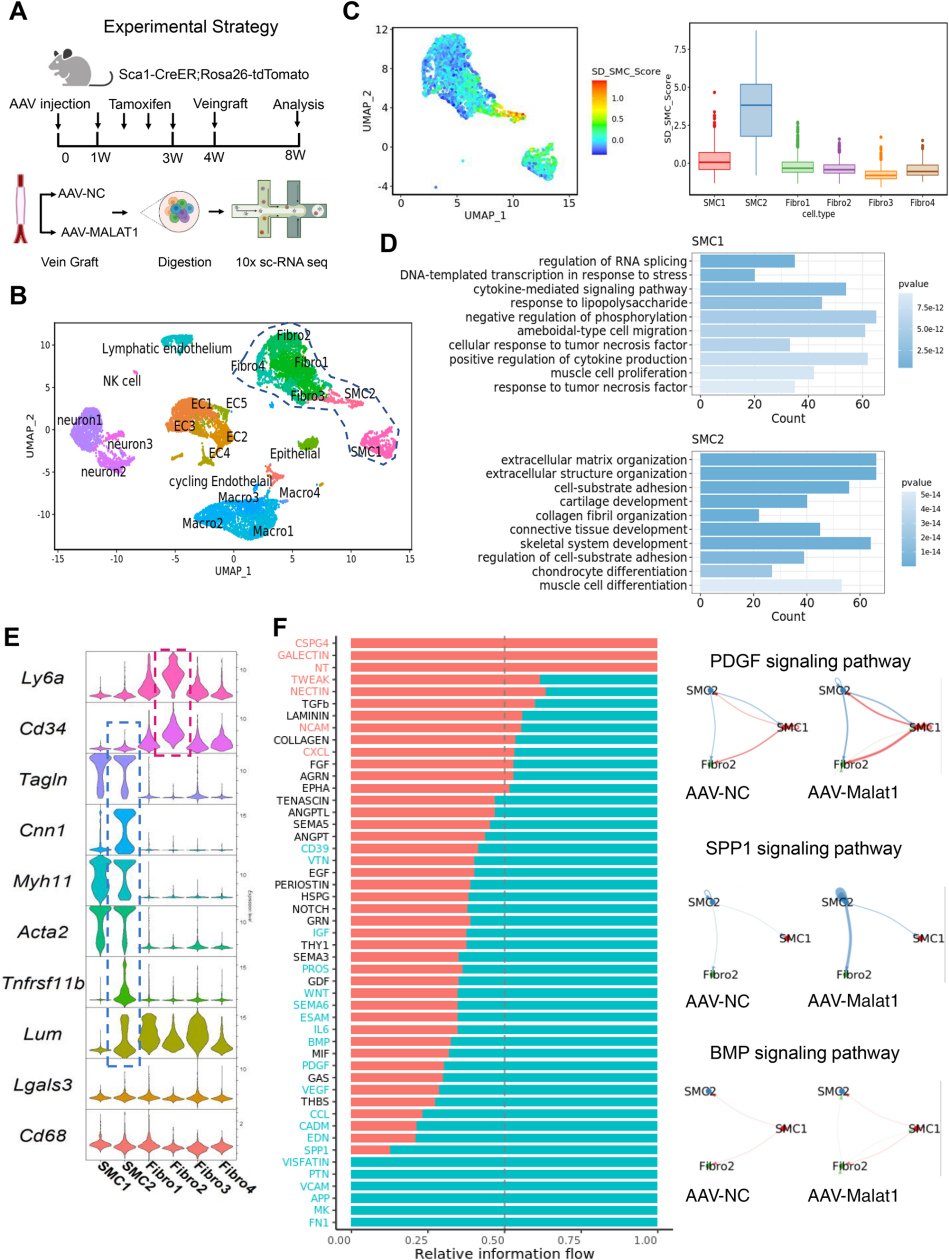
ScRNA seq maps SD_SMCs back to whole-cell analysis and elucidate that Malat1 loss alters cell-to-cell interactions. **A** Schematic diagram showing the experimental strategy of animal experiments and single cell sequencing. **B** Umap distribution of all cell types obtained from mouse veingraft in AAV-NC and AAV-Malat1 groups, *n* = 6 for each group. Clusters which were distributed close to SMCs were selected for downstream analysis. **C** A SD_SMC score was calculated based on the expression of top differentially expressed genes between SD_SMC (from Fig. 5B) and other clusters. The red color indicates high similarity to SD_SMC. A boxplot (right panel) showing the SD_SMC score for the clusters selected from panel **A**. **D** GO biological pathway analysis of the top differentially expressed genes in SMC1 and SMC2. Top 10 enriched pathways are presented. **E** A violinplot showing the canonical cell markers of stem/progenitor cells (Ly6a, Cd34), SMCs (Tagln, Cnn1, Acta2) macrophage-like SMCs (Lgals3, Cd68), and highly-expressed markers of SD_SMCs (Tnfrsf11b, Lum). **F** Heatmap illustration of the cell chat signaling pathway between SMC1, SMC2 and Fibro2 of AAV-NC (red) and AAV-Malat1 (blue) groups. Circle plot showing PDGF, SPP1 and BMP pathways. The arrows emerging from cells indicate ligands, and the cells pointed by the arrows are the receptors. The thicker the line, the more ligand-receptor pairs are activated

We also performed whole-cell scRNA sequencing of the mice veingraft. The AAV-NC and AAV-Malat1 groups were treated separately (Fig. 6A). After quality control, 7249 cells from the AAV-Malat1 group and 6251 cells from the AAV-NC group were collected. Figure 6B shows the Umap clustering of the combined data of the two groups. We wondered whether SD_SMCs could be distinguished from normal SMCs. Therefore, an SD_SMC score was computed based on the expression of the top differentially expressed genes between SD_SMC and other clusters (from Fig. 5B). Feature plot displayed the SD_SMC score in clusters juxtaposed in Umap space to the SMC clusters. The box plot demonstrated more clearly that the SD_SMC score was higher in SMC2 (Fig. 6C). To investigate the difference between SMC1 and SMC2, GO analysis was performed based on top-expressing genes. The results showed that SMC2 was more related to muscle cell differentiation (Fig. 6D). In Fig. 6E, the violin plot of markers in Fig. 5E further confirmed that SMC2 could be mapped back to SD_SMCs. In the meantime, “fibro2” cluster shared a high degree of similarity with the SPCs depicted in Fig. 5. Then we performed cell chat analysis to examine how cell-to-cell interactions changed after knocking down Malat1. The heatmap (Fig. 6F) showed an overall alteration in the cell interaction network. Blue signal pathways were upregulated in AAV-Malat1 group. PDGF signaling pathway, which plays a critical role in smooth muscle development and phenotype transition [37, 38], is upregulated in Malat1-knockdown group. *SPP1* signaling pathway [39], which was reported to regulate cancer cell proliferation and migration was also up-regulated. BMP signaling pathway also plays an important role in regeneration of SMC and the maintenance of the contractile state [40]. In addition, *Midkine* (*MK*) and *pleiotrophin* (*PTN*), which are members of the subfamily of heparin-binding growth factors, have been shown to play a role in cell differentiation and angiogenesis [41, 42]. Taken together, these findings show that SD_SMCs could be distinguished from normal SMCs even at low resolution, and Malat1 alters intercellular signaling pathways involved in the control of SMC differentiation and vasculature.

### The role of Malat1 in Sca1^+^ SPCs’ differentiation into SMCs in vitro

**Fig. 7:**
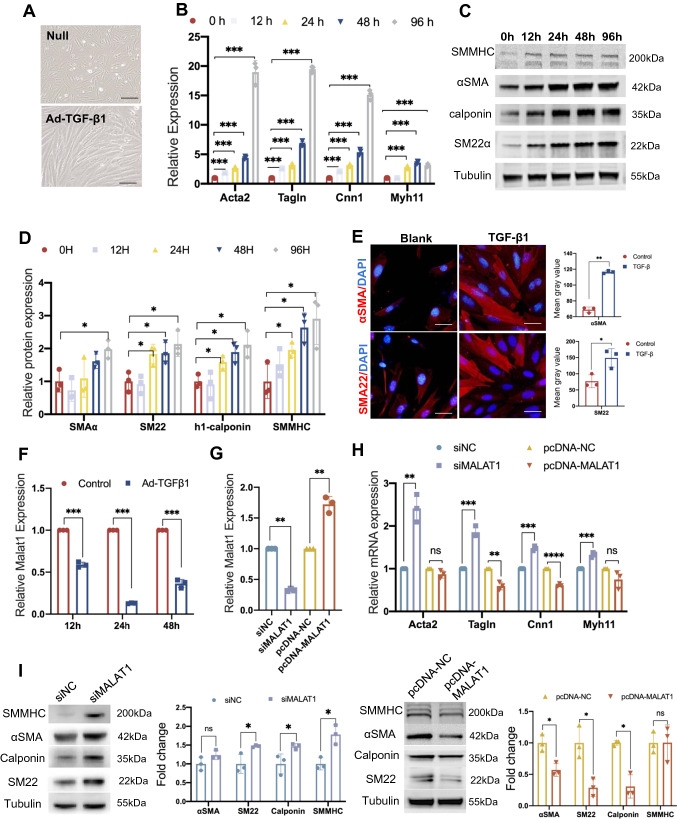
The role of Malat1 in Sca1^+^ SPCs’ differentiation into SMCs in vitro. **A** Morphological changes in Sca1^+^ SPCs following 10 ng/ml TGF-β1 treatment for 24 h. Scale bars, 50 μm. **B**–**D** The mRNA or protein expression level of αSMA, SM22, Calponin, and SMMHC as determined by RT-PCR (**B**) or Western blotting assay (**C**) at different time points (0 h, 12 h, 24 h, 48 h, 96 h) after culture with TGF-β1. Protein bands were quantified by densitometry and normalized to the density of Tubulin (**D**). Data are shown as the Mean ± SD. **E** Immunofluorescence staining of αSMA and SM22 in cells treated with or without TGF-β1 for 72 h. Scale bars, 20 μm. The right panel shows the quantification of fluorescence intensity value. **F** The RNA expression level of Malat1 during differentiation process as determined by RT-PCR assay. **G** RT-PCR test results showing the expression level of Malat1 in Sca1^+^ SPCs after transfection with siNC/siMalat1 or pcDNA-NC/pcDNA-Malat1. **H**, **I** Expression level of αSMA, SM22, Calponin, and SMMHC in Sca1^+^ SPCs transfected with siNC/siMalat1 or pcDNA-NC/pcDNA-Malat1 and cultured in TGF-β1 as measured using RT-PCR (**H**) and Western blotting (**I**). Bands were quantified by densitometry and normalized to the density of Tubulin. Data are shown as the mean ± SD. ****P < 0.0001, ***P < 0.001, **P < 0.01, *P < 0.05, ^ns^*P* ≥ 0.05

Previous research has shown that PDGF-BB [10], Type IV collagen [43], and TGF-β1 [44, 45] can induce Sca1^+^ to differentiate into SMCs. TGF-β1 has also been shown to contribute to the development of SMCs from embryonic stem cells via Smad2 and Smad3 pathways [46]. Herein, we investigated the intercellular signaling patterns of SPCs, SD_SMCs, Fibro1, and Fibro2 (Fig. S4B), and discovered that Collagen, PDGF, and TGFβ pathways were highly active (Fig. S4C). Therefore, we stimulated Sca1^+^ SPCs with Type IV collagen (Fig. S5A), PDGF-BB (Fig. S5B), and TGF-β1 and concluded that TGF-β1 could promote Sca1^+^ SPCs differentiation into SMCs with the highest efficacy. To begin with, the morphological changes of Sca1^+^ SPCs treated with TGF-β1 for 24 h were very obvious in our observations. Figure 7A shows that the cell shape changed from short ellipses to fusiform. Sca1^+^ SPCs were subsequently cultured with TGF-β1 for 0, 12, 24, 48, and 96 h, respectively at 37 °C, before being harvested to detect the expression of SMC markers, including *Acta2*, *Tagln*, *Cnn1* (encoding Calponin1), and *Myh11*. RT-PCR revealed significant up-regulation of these markers at the mRNA level after stimulation with TGF-β1 (Fig. 7B). Western blotting confirmed the up-regulation at the protein level, which is consistent with mRNA (Fig. 7C, D). Immunofluorescence staining also showed that the fluorescence intensity of αSMA and SM22 increased in Sca1^+^ SPCs cultured with TGF-β1 for 3 days (Fig. 7E). These findings elucidate that Sca1^+^ SPCs can repopulate SMCs in vitro.

Interestingly, the expression of Malat1 was significantly decreased during the differentiating process (Fig. 7F). We investigated further the function of Malat1 in Sca1^+^ SPCs by silencing Malat1 expression with siRNAs and overexpressing Malat1 by plasmids. (Fig. 7G). Malat1 silencing increased the expression of αSMA, SM22, Calponin, and SMMHC at both the mRNA and protein levels, while Malat1 overexpression decreased SMC markers to different extends. (Fig. 7H, I). These findings demonstrated that Malat1 is a critical regulator of Sca1^+^ SPCs’ differentiation into SMCs in vitro by altering the expression of contractile proteins.

### Malat1 regulates Sca1^+^ SPCs’ differentiation into SMCs via Malat1/miR125a-5p/Stat3 signaling pathway

**Fig. 8:**
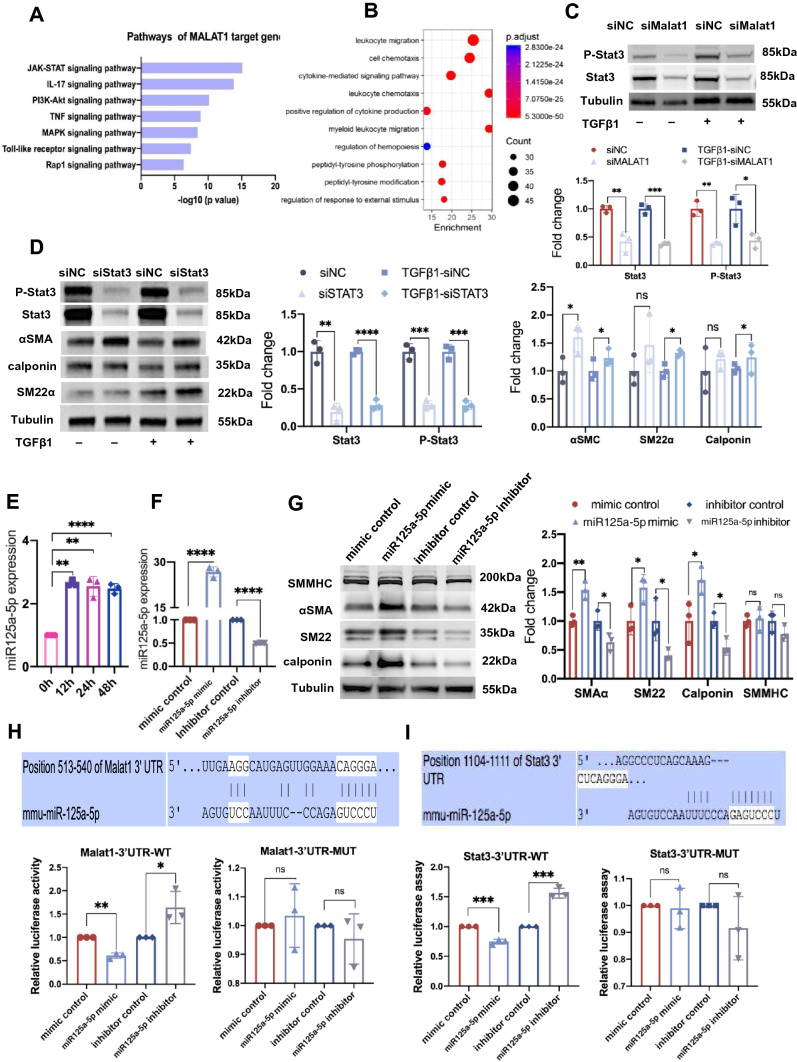
Malat1 regulates Sca1^+^ SPCs’ differentiation into SMCs via Malat1/miR125a-5p/Stat3 signaling pathway. **A** Seven representative pathways from the top 20 KEGG enriched pathways of DEPs in Fig. S4A. **B** Top 10 enriched GO Biological Process terms of DEPs. **C** Western blotting demonstrating the effect of Malat1 deficiency on the Stat3 signaling pathway. The density level of cleaved p-Stat3 and Stat3 was normalized to the density of Tubulin. **D** Western blotting showing the protein expression level of P-Stat3, Stat3, αSMA, SM22, Calponin and Tubulin in each group. Protein bands were quantified by densitometry and normalized to the density of Tubulin. **E** mRNA expression level of miR-125a-5p as quantified by RT-PCR at different time points after cultured with TGF-β1. **F** RT-PCR results showing the expression level of miR-125a-5p in Sca-1^+^cells after transfection with mimic control/miR-125a-5p mimic and inhibitor control/miR-125a-5p inhibitor. **G** Western blotting results showing the expression level of SMC markers in Sca-1^+^cells after transfected with mimic control/miR-125a-5p mimic and inhibitor control/miR-125a-5p inhibitor and cultured with TGF-β1. **H** The firefly luciferase and renilla luciferase activity of Sca-1^+^ cells co-transfected with Malat1-WT-3′UTR or Malat1-MUT-3′UTR and mimic control/miR-125a-5p mimic/inhibitor control/miR-125a-5p inhibitor. **I** The firefly luciferase and renilla luciferase activity of Sca-1^+^ cells co-transfected with Stat3-WT-3′UTR or Stat3-MUT-3′UTR and mimic control/miR-125a-5p mimic/inhibitor control/miR-125a-5p inhibitor. Data are shown as the mean ± SD. ****P < 0.0001, ***P < 0.001, **P < 0.01, *P < 0.05, ^ns^*P* ≥ 0.05

To gain further insight into the mechanism by which Malat1 modulates Sca1^+^ SPCs’ differentiation, we performed protein array chip (including 400 targets) sequencing on Sca1^+^ cells transfected with siMalat1 and treated with TGF-β1 in vitro. 132 differentially expressed proteins (DEPs) were identified as having a foldchange greater than 1.2 or less than 0.83 (absolute logFC > 0.263) (Fig. S4A). DEPs were then subjected to the KEGG (Kyoto Encyclopedia of Genes and Genomes) pathway and GO enrichment analysis. Figure 8A shows the 7 representative pathways from the top 20 KEGG enriched pathways of DEPs, including JAK-STAT, IL-17, PI3K-Akt, MAPK, and Rap1 (Ras-related protein 1) signaling pathways, which are known to be required for normal vasculature development and angiogenesis. Figure 8B shows the top 10 biological processes enriched in GO analysis, including ‘cell chemotaxis’, ‘positive regulation of cytokine production’ and ‘positive regulation of response to external stimulus’ etc., indicating that Sca1^+^ SPCs in the Malat1 deficiency group were more responsive, which was consistent with the scRNA sequencing results. Since JAK/STAT was the most actively regulated pathway and Malat1 was reported to be associated with Stat3 [47], we hypothesized that Malat1 modulates Sca1^+^ SPCs’ differentiation via Stat3 signaling. Therefore, we performed a western blot analysis and discovered that knocking down Malat1 inhibited Stat3 and p-Stat3 with or without TGF-β1 treatment (Fig. 8C), indicating that Stat3 may be a prospective candidate. Then we designed siRNA targeting Stat3 and verified its efficacy by western blotting. To our surprise, knocking down Stat3 in Sca1^+^ SPCs increased the expression of αSMA, SM22 and Calponin after TGF-β1 treatment (Fig. 8D).

**Fig. 9:**
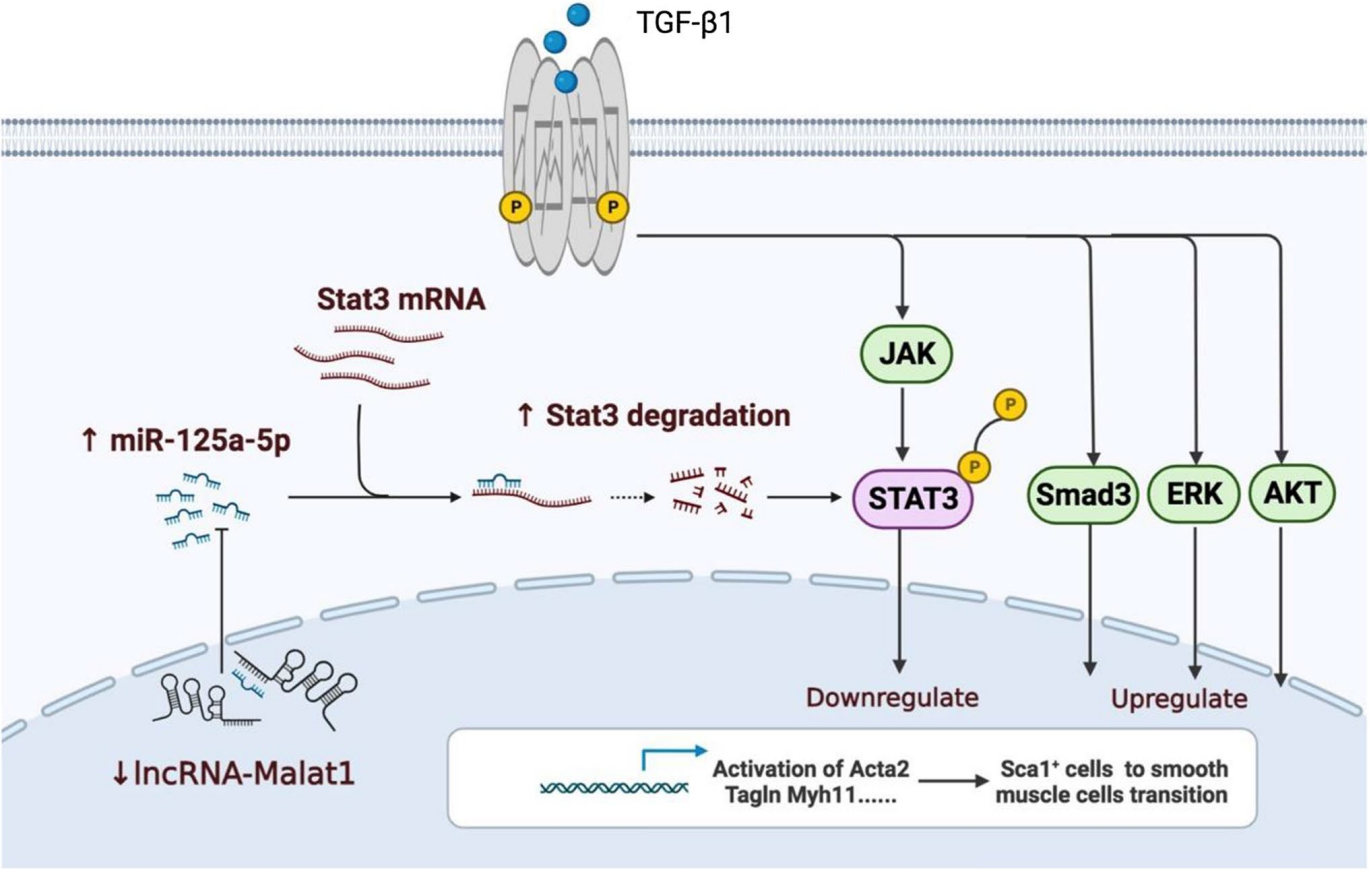
Sketch map of Malat1-miR125a-5p-Stat3 pathway that regulates Sca1^+^ cells’ transition into SMCs with the treatment of TGF-β1

But how does Malat1 regulate Stat3? It is commonly accepted that lncRNAs exert regulatory effects through different mechanisms, the most prominent one is interacting with miRNAs. Therefore, we investigated whether Malat1 sponge microRNAs to alter Stat3 expression. To address this, candidate miRNAs targeting Malat1 and Stat3 were predicted using TargetScan. We found that miR-106b-5p, miR-20b-5p, miR-17-5p and miR-125a-5p were most closely related to Malat1 and Stat3. Further RT-PCR assays confirmed miR-106b-5p, miR-20b-5p and miR-17-5p could not regulate Sca1^+^ to differentiate into SMCs effectively (Fig. S5C–E) and miR-125a-5p was the candidate miRNA. RT-PCR results indicated that miR-125a-5p expression levels were significantly upregulated in TGF-β1 stimulated Sca1^+^ cells (Fig. 8F), which is the opposite of TGF-β1’s effect on Malat1. Furthermore, overexpression of miR-125a-5p by transfecting its mimics appears to stimulate the expression of *Acta, Tagln, Cnn1*, and *Myh11*, whereas miR-125a-5p inhibitors suppressed it (Fig. 8G). To confirm the interaction between miR-125a-5p and Malat1 or Stat3, we predicted the miR-125a-5p-binding sites in the 3′-UTR of Malat1 or Stat3, and constructed luciferase reporter plasmids containing either wild-type or mutant 3′-UTR binding sites of Malat1 and Stat3, respectively. Co-transfection of Malat1-3′-UTR-WT and miR-125a-5p mimics into Sca1^+^ cells resulted in a significant reduction in luciferase activity than co-transfection with the mimic control. In cells transfected with the miR-125a-5p inhibitors, luciferase activity was increased. Co-transfection of Malat1-3′-UTR-MUT with miR-125a-5p mimics or inhibitors did not affect luciferase activity (Fig. 8H). Similar results were also observed in Stat3 (Fig. 8I). Although additional research is needed, our findings imply that Malat1 may function as a sponge for miR-125a-5p to regulate Stat3 expression, suppressing the Sca1^+^ SPC to SMC transition (Fig. 9).

## Discussion

In this study, we used genetic lineage tracing to demonstrate that the dynamic balance of Sca1-SMC transition is essential for vascular remodeling. Excessive SMC accumulation derived from Sca1^+^ SPCs may result in stenosis and thrombosis. Ablation of Sca1^+^ cells attenuated venous arterialization and vascular structure normalization during vessel repair and regeneration. Our findings provide new cellular and molecular perspectives on hemodialysis vascular access dysfunction and other human vascular pathologies, including atherosclerosis and angioplasty-induced vessel injury. In the context of hemodialysis fistula, previous research has demonstrated that SMC converts to a synthetic phenotype, hence contributing to fistula failure. Increased expression of cytokines such as TGF-β1 and IGF-I was observed in failed fistula, and it was hypothesized that these cytokines stimulated SMC proliferation and extracellular matrix (ECM) synthesis [48]. However, the study lacked in vivo evidence. Others hypothesized that SMC migrated from media to the intima and then proliferated to form neointima [49]. However, the majority of this information is derived from arterial angioplasty models rather than from vascular (venous) anastomosis models. Zhao et al*.* performed AVF surgery on *Myh11-CreER* SMC lineage-tracing mice and hypothesized that pre-existing SMC contributed to venous media thickening, but found it only contributed half of SMCs in neointima hyperplasia, thereby demonstrating that resident progenitor cells are an essential component of SMCs regeneration [8]. Using the same SMC lineage-tracing mice, Roostalu et al*.* demonstrated that the fraction of SMCs originating from pre-existing SMCs was significantly diluted following severe transmural injury and replaced by adventitial cells expressing markers such as Sca1^+^, CD44^+^, and CD34^+^ [11]. Consequently, a recent study utilized *Sca1-CreER* lineage tracing and provided direct evidence that adventitial Sca1^+^ progenitors differentiate into de novo SMCs in trans-sectionally injured femoral arteries [12]. Interestingly, using single-cell sequencing, a previous study detected a population of Sca1^+^ SMCs in both healthy mouse vessels and atherosclerotic plaques. They further observed that this subset represented a more plastic state, ready to respond to injury or inflammation. Transcriptomic analysis revealed that these Sca1^+^ cells may originate from phenotypically modulated SMCs [50]. All these data together with our findings demonstrated that there may exist a dynamic balance between Sca1^+^SPCs and SMCs. In homeostasis, Sca1^+^ SPCs, and SMCs compensate for SMCs loss during daily wear and tear, and SMCs may de-differentiate to maintain normal function. While SMCs are insufficient to fuel the smooth muscle pool after severe transmural injury, Sca1^+^SPCs are activated and aggressively generate a mass of SMCs, potentially causing extensive neointima hyperplasia and stenosis. However, this required further verification using dual-lineage tracing models to trace the trajectories of Sca1^+^ cells and SMCs.

In this study, we also gained insight into the transcriptional phenotype of Sca1^+^ SPC-derived SMCs. Tang et al*.* discovered that the number of Sca1-derived SMCs is larger than pre-existing SMC-derived SMCs using a flow cytometry assay, and proposed that Sca1-derived SMC expands more than pre-existing SMCs during vessel recovery, but didn’t analyze its transcriptional profile. Herein, we performed scRNA seq and demonstrated that Sca1-derived SMCs had a fibroblast-like phenotype. The role of fibroblast-like SMCs has recently been proposed and discussed. Wirka et al*.* [36] reported that in atherosclerotic lesions, SMCs transform into fibroblast-like cells (termed fibromyocytes) in vivo in both human and mice, which also highly express *Tnfrsf11b*. However, they also discovered that many modulated SMCs within the lesion express Sca-1 [50] and stated that it is undetermined if these fibromyocytes originated from these cells. Based on these findings, it is not possible to determine whether fibroblast-like SMCs originate from the transdifferentiation of pre-existing SMCs or the differentiation of Sca1^+^ cells. Nonetheless, the existence of fibromyocytes was confirmed and the transcriptional profile of fibromyocytes in the mouse might be used to identify an orthologous human fibromyocyte population in a variety of vascular diseases.

Our group proposed that Sca1^+^ cells in the adventitial layer of the vessel wall are stem cells for smooth muscle and initially cultured them in vitro. When stimulated in vitro with platelet-derived growth factor-BB (PDGF-BB), isolated Sca1^+^ cells could differentiate into SMCs [10]. In addition, Type IV collagen and TGF-β1 have been established to induce Sca1^+^ to differentiate into SMCs [43–45]. Moreover, TGF-β1 is closely related to AVF maturation and dysfunction [48]. It is upregulated in failed fistula and may contribute to a sex difference in AVF. Female mice with a higher TGF-β1 expression had a smaller venous diameter and an increased neointima area-to-media ratio following the AVF procedure [51]. Therefore, we chose TGF-β1 for our in vitro experiments, to induce Sca1^+^ SPCs into SMCs with high efficacy. This could pave the way for the in vitro study of other smooth muscle progenitors.

We also confirmed that lncRNA Malat1 is a critical element for modulating Sca1^+^SPCs’ differentiation towards SMCs both in vivo and in vitro. Malat1 has been extensively studied in cardiovascular diseases. Initially, most studies focused on its function in the context of the systemic vasculature and endothelial cells [22, 52, 53]. Following this, an increasing number of studies investigating its role in smooth muscle cells emerged. A study of pulmonary hypertension found that the level of Malat1 was significantly increased in hypoxic human pulmonary artery SMCs, and Malat1 depletion inhibited SMC migration and proliferation [23]. In addition, Christian et al*.* revealed that Malat1 disruption inhibits experimental aneurysm growth by restoring contractile protein expression in SMCs and improving aortic mural architecture [54]. Another study reported that Malat1 promotes the transformation of SMCs from contraction to synthetic phenotypes [55]. Recently, Yu et al*.* demonstrated that Malat1 knockout could alleviate the reduction of the SMC contractile phenotype and exert preventive, inhibitory, and reversal effects on Angiotensin II-induced abdominal aortic aneurysm [56]. Taken together, Malat1 seemed to be associated with SMCs’ loss of contractile phenotype. In this study, Malat1 was for the first time investigated in vascular stem cells. Notably, we discovered that Malat1 reduction can promote Sca1^+^ SPCs to transform into SMCs in vivo, which is consistent with previous research. We also found that Sca1^+^ cells deletion resulted in decreased SMC regeneration and restored Malat1 expression. As mentioned above, Malat1 expression in SMCs and SPCs was suppressed during vascular remodeling diseases including AVF, AS and artery injury. But when Sca1^+^ cells were ablated, SMC regeneration and related signal pathways were not fully activated, thus, resulting in inadequate Malat1 suppression compared to the control group. Together with our in vitro experiments, these studies demonstrated that Malat1 could suppress contractile proteins expression in SMCs and Sca1^+^ SPCs. In diseases that SMCs regenerate like vascular injury and atherosclerosis, Malat1 expression level is down-regulated; while in diseases that SMCs are reduced or lost contractile phenotype, Malat1 is up-regulated. Further in vitro experiments demonstrated that Malat1 regulated the expression of contractile proteins via miR125a-5p/Stat3 signaling pathway.

It is now generally accepted that vascular resident progenitor cells are significant sources of SMCs and endothelial cells. They are crucial for maintaining and regenerating the vessel wall. In the present study, we sought to provide new cellular and molecular perspectives on human AVF maturation and stenosis, identifying lncRNA Malat1 as a potent regulator. However, because the regulatory network is complex, additional mechanism research is needed. Altogether, these evidence of SMC generation from Sca1^+^ SPCs via Malat1 provides new insights and raises new concerns regarding the cellular and molecular mechanisms underlying vascular disease and regeneration.

## Supplementary Information

Below is the link to the electronic supplementary material.

Supplementary file1 (PDF 3443 KB)

Supplementary file2 (DOCX 25 KB)

## Data Availability

The data and R scripts that related to the findings of this study are available on reasonable request. ScRNA-seq data of this study are available in Gene Expression Omnibus (GSE226135).

## References

[1] Vascular Access Work Group (2006). Clinical practice guidelines for vascular access. Am J Kidney Dis.

[2] Allon M (2019). Vascular access for hemodialysis patients: new data should guide decision making. Clin J Am Soc Nephrol.

[3] Feldman HI, Kobrin S, Wasserstein A (1996). Hemodialysis vascular access morbidity. J Am Soc Nephrol.

[4] Roy-Chaudhury P, Sukhatme VP, Cheung AK (2006). Hemodialysis vascular access dysfunction: a cellular and molecular viewpoint. J Am Soc Nephrol.

[5] Masud A (2018). The complications of vascular access in hemodialysis. Semin Thromb Hemost.

[6] Motwani JG, Topol EJ (1998). Aortocoronary saphenous vein graft disease: pathogenesis, predisposition, and prevention. Circulation.

[7] Hu Y (2002). Both donor and recipient origins of smooth muscle cells in vein graft atherosclerotic lesions. Circ Res.

[8] Zhao J (2017). Dual function for mature vascular smooth muscle cells during arteriovenous fistula remodeling. J Am Heart Assoc.

[9] Tang Z (2012). Differentiation of multipotent vascular stem cells contributes to vascular diseases. Nat Commun.

[10] Hu Y (2004). Abundant progenitor cells in the adventitia contribute to atherosclerosis of vein grafts in ApoE-deficient mice. J Clin Investig.

[11] Roostalu U (2018). Distinct cellular mechanisms underlie smooth muscle turnover in vascular development and repair. Circ Res.

[12] Tang J (2020). Arterial Sca1(+) vascular stem cells generate de novo smooth muscle for artery repair and regeneration. Cell Stem Cell.

[13] Hutchinson JN (2007). A screen for nuclear transcripts identifies two linked noncoding RNAs associated with SC35 splicing domains. BMC Genom.

[14] Ji P (2003). MALAT-1, a novel noncoding RNA, and thymosin beta4 predict metastasis and survival in early-stage non-small cell lung cancer. Oncogene.

[15] Li L (2009). Role of human noncoding RNAs in the control of tumorigenesis. Proc Natl Acad Sci USA.

[16] Ji Q (2014). Long non-coding RNA MALAT1 promotes tumour growth and metastasis in colorectal cancer through binding to SFPQ and releasing oncogene PTBP2 from SFPQ/PTBP2 complex. Br J Cancer.

[17] Bi S (2017). LncRNA-MALAT1-mediated Axl promotes cell invasion and migration in human neuroblastoma. Tumour Biol.

[18] Li P (2017). MALAT1 Is associated with poor response to oxaliplatin-based chemotherapy in colorectal cancer patients and promotes chemoresistance through EZH2. Mol Cancer Ther.

[19] Tripathi V (2013). Long noncoding RNA MALAT1 controls cell cycle progression by regulating the expression of oncogenic transcription factor B-MYB. PLoS Genet.

[20] Wu XS (2014). MALAT1 promotes the proliferation and metastasis of gallbladder cancer cells by activating the ERK/MAPK pathway. Cancer Biol Ther.

[21] Shen L (2015). Long noncoding RNA MALAT1 promotes brain metastasis by inducing epithelial-mesenchymal transition in lung cancer. J Neurooncol.

[22] Michalik KM (2014). Long noncoding RNA MALAT1 regulates endothelial cell function and vessel growth. Circ Res.

[23] Brock M (2017). Analysis of hypoxia-induced noncoding RNAs reveals metastasis-associated lung adenocarcinoma transcript 1 as an important regulator of vascular smooth muscle cell proliferation. Exp Biol Med (Maywood).

[24] Cremer S (2019). Hematopoietic deficiency of the long noncoding RNA MALAT1 promotes atherosclerosis and plaque inflammation. Circulation.

[25] Jiang L (2021). Nonbone marrow CD34(+) cells are crucial for endothelial repair of injured artery. Circ Res.

[26] Tang X (2019). Molecular mechanisms involved in TGF-beta1-induced muscle-derived stem cells differentiation to smooth muscle cells. Am J Transl Res.

[27] Ni Z (2019). Recipient c-kit lineage cells repopulate smooth muscle cells of transplant arteriosclerosis in mouse models. Circ Res.

[28] van Kuijk K (2019). Heterogeneity and plasticity in healthy and atherosclerotic vasculature explored by single-cell sequencing. Cardiovasc Res.

[29] Xu Q (2004). Mouse models of arteriosclerosis: from arterial injuries to vascular grafts. Am J Pathol.

[30] Schwartz SM, de Blois D, Obrien ER (1995). The intima. Soil for atherosclerosis and restenosis. Circ Res.

[31] Basatemur GL (2019). Vascular smooth muscle cells in atherosclerosis. Nat Rev Cardiol.

[32] Buch T (2005). A Cre-inducible diphtheria toxin receptor mediates cell lineage ablation after toxin administration. Nat Methods.

[33] Naglich JG (1992). Expression cloning of a diphtheria toxin receptor: identity with a heparin-binding EGF-like growth factor precursor. Cell.

[34] Kleefeldt F (2022). Bone marrow-independent adventitial macrophage progenitor cells contribute to angiogenesis. Cell Death Dis.

[35] Shankman LS (2015). KLF4-dependent phenotypic modulation of smooth muscle cells has a key role in atherosclerotic plaque pathogenesis. Nat Med.

[36] Wirka RC (2019). Atheroprotective roles of smooth muscle cell phenotypic modulation and the TCF21 disease gene as revealed by single-cell analysis. Nat Med.

[37] Heusch G (2014). Cardiovascular remodelling in coronary artery disease and heart failure. Lancet.

[38] Gomez D, Owens GK (2012). Smooth muscle cell phenotypic switching in atherosclerosis. Cardiovasc Res.

[39] Deng G (2020). BET inhibitor suppresses melanoma progression via the noncanonical NF-kappaB/SPP1 pathway. Theranostics.

[40] Wang L (2021). BMP9 and BMP10 act directly on vascular smooth muscle cells for generation and maintenance of the contractile state. Circulation.

[41] Muramatsu T (2010). Midkine, a heparin-binding cytokine with multiple roles in development, repair and diseases. Proc Jpn Acad Ser B Phys Biol Sci.

[42] Xu C (2014). Functional receptors and intracellular signal pathways of midkine (MK) and pleiotrophin (PTN). Biol Pharm Bull.

[43] Xiao Q (2007). Stem cell-derived Sca-1+ progenitors differentiate into smooth muscle cells, which is mediated by collagen IV-integrin alpha1/beta1/alphav and PDGF receptor pathways. Am J Physiol Cell Physiol.

[44] Sainz J (2006). Isolation of "side population" progenitor cells from healthy arteries of adult mice. Arterioscler Thromb Vasc Biol.

[45] Hirschi KK, Rohovsky SA, D'Amore PA (1998). PDGF, TGF-beta, and heterotypic cell-cell interactions mediate endothelial cell-induced recruitment of 10T1/2 cells and their differentiation to a smooth muscle fate. J Cell Biol.

[46] Sinha S (2004). Transforming growth factor-beta1 signaling contributes to development of smooth muscle cells from embryonic stem cells. Am J Physiol Cell Physiol.

[47] Dai Q, Zhang T, Li C (2020). LncRNA MALAT1 regulates the cell proliferation and cisplatin resistance in gastric cancer via PI3K/AKT pathway. Cancer Manag Res.

[48] Stracke S (2002). Increased expression of TGF-beta1 and IGF-I in inflammatory stenotic lesions of hemodialysis fistulas. Kidney Int.

[49] Roy-Chaudhury P (2003). Hemodialysis vascular access dysfunction: from pathophysiology to novel therapies. Blood Purif.

[50] Dobnikar L (2018). Disease-relevant transcriptional signatures identified in individual smooth muscle cells from healthy mouse vessels. Nat Commun.

[51] Cai C (2020). Differences in transforming growth factor-beta1/BMP7 signaling and venous fibrosis contribute to female sex differences in arteriovenous fistulas. J Am Heart Assoc.

[52] Puthanveetil P (2015). Long non-coding RNA MALAT1 regulates hyperglycaemia induced inflammatory process in the endothelial cells. J Cell Mol Med.

[53] Wu Q (2020). Extracellular vesicles from human embryonic stem cell-derived cardiovascular progenitor cells promote cardiac infarct healing through reducing cardiomyocyte death and promoting angiogenesis. Cell Death Dis.

[54] Lino Cardenas CL (2018). An HDAC9-MALAT1-BRG1 complex mediates smooth muscle dysfunction in thoracic aortic aneurysm. Nat Commun.

[55] Song TF (2018). LncRNA MALAT1 regulates smooth muscle cell phenotype switch via activation of autophagy. Oncotarget.

[56] Yu L (2022). An intersegmental single-cell profile reveals aortic heterogeneity and identifies a novel Malat1(+) vascular smooth muscle subtype involved in abdominal aortic aneurysm formation. Signal Transduct Target Ther.

