# Supplementary material for: Fate mapping RNA-sequencing reveal Malat1 regulates Sca1+ progenitor cells to vascular smooth muscle cells transition in vascular remodeling
Source: Cell Mol Life Sci. 2023 Apr 6;80(5):118. doi: 10.1007/s00018-023-04762-3 (PMC10079726; doi:10.1007/s00018-023-04762-3)
Supplement: Supplementary file 1 — Supplementary file1 (PDF 3443 KB) [file 18_2023_4762_MOESM1_ESM.pdf]

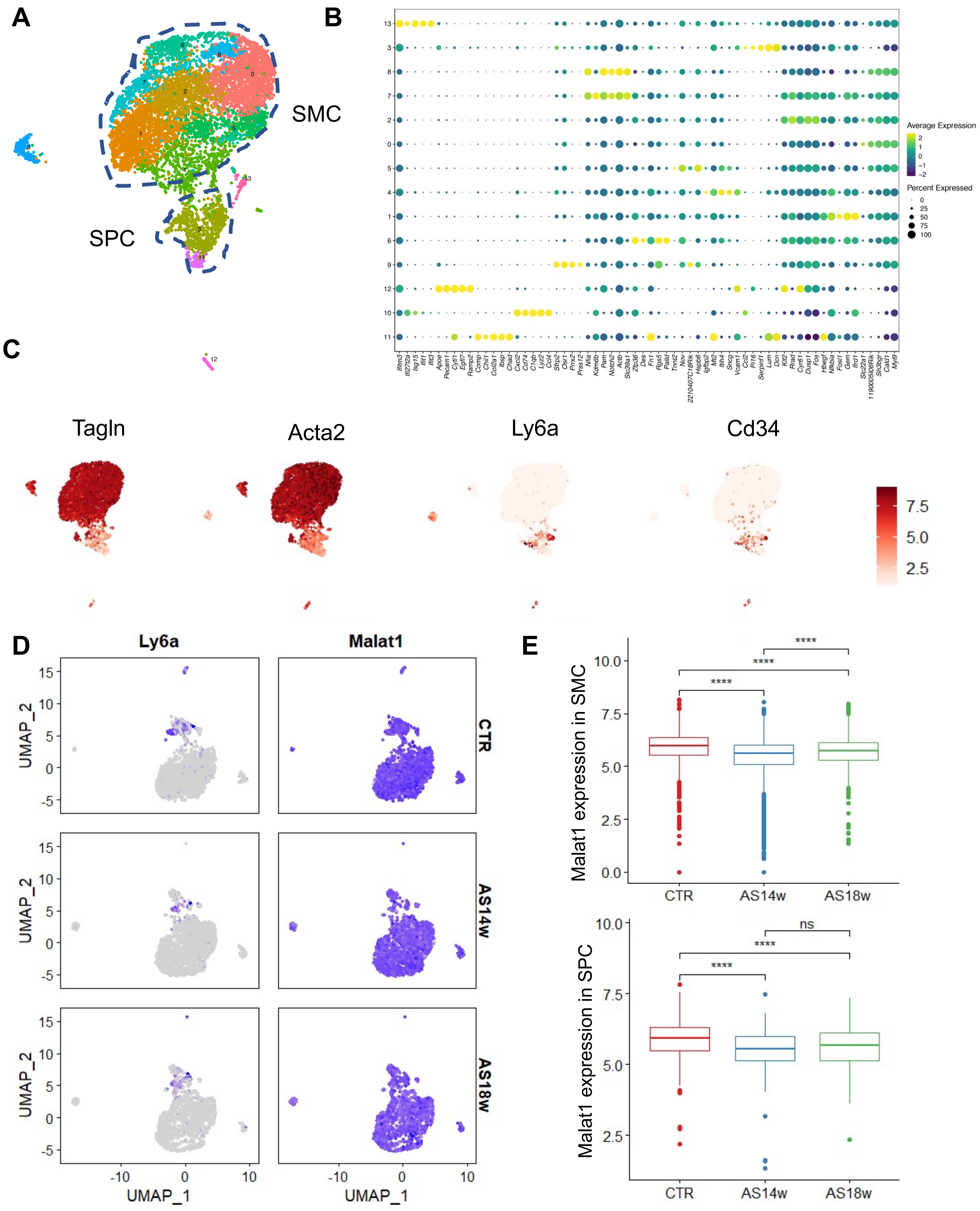
A
B
C
D
E
SMC
SPC
Average Expression
Percent Expressed
Tagln
Acta2
Ly6a
Cd34
7.5
5.0
2.5
Ly6a
Malat1
CTR
AS14w
AS18w
UMAP_2
UMAP_1
Malat1 expression in SMC
Malat1 expression in SPC
****
ns
CTR
AS14w
AS18w

Fig. S1 The expression of Malat1 declined in SMCs and $Sca1^+$ SPCs from atherosclerotic plaques.

A, Umap visualization of cell types present in the aortic plaques of mice fed on a cholesterol-rich diet for 14 or 18 weeks and control group. After the quality control process, 7536 cells were selected for downstream analysis. The cells were grouped into mainly two clusters; one enriched with SMCs and another with SPCs. **B**, A dot plot showing the top 5 genes defining each cluster. **C**, Expression of Tagln, Acta2, Ly6a, and Cd34 displayed on Umap visualization. **D**, Expression level of Ly6a and Malat1 in each group. **E**, Box plot showing Malat1 expression levels in SMCs and SPCs of each group. P value was calculated with unpaired t test. **** $P < 0.0001$, ns $P \geq 0.05$.

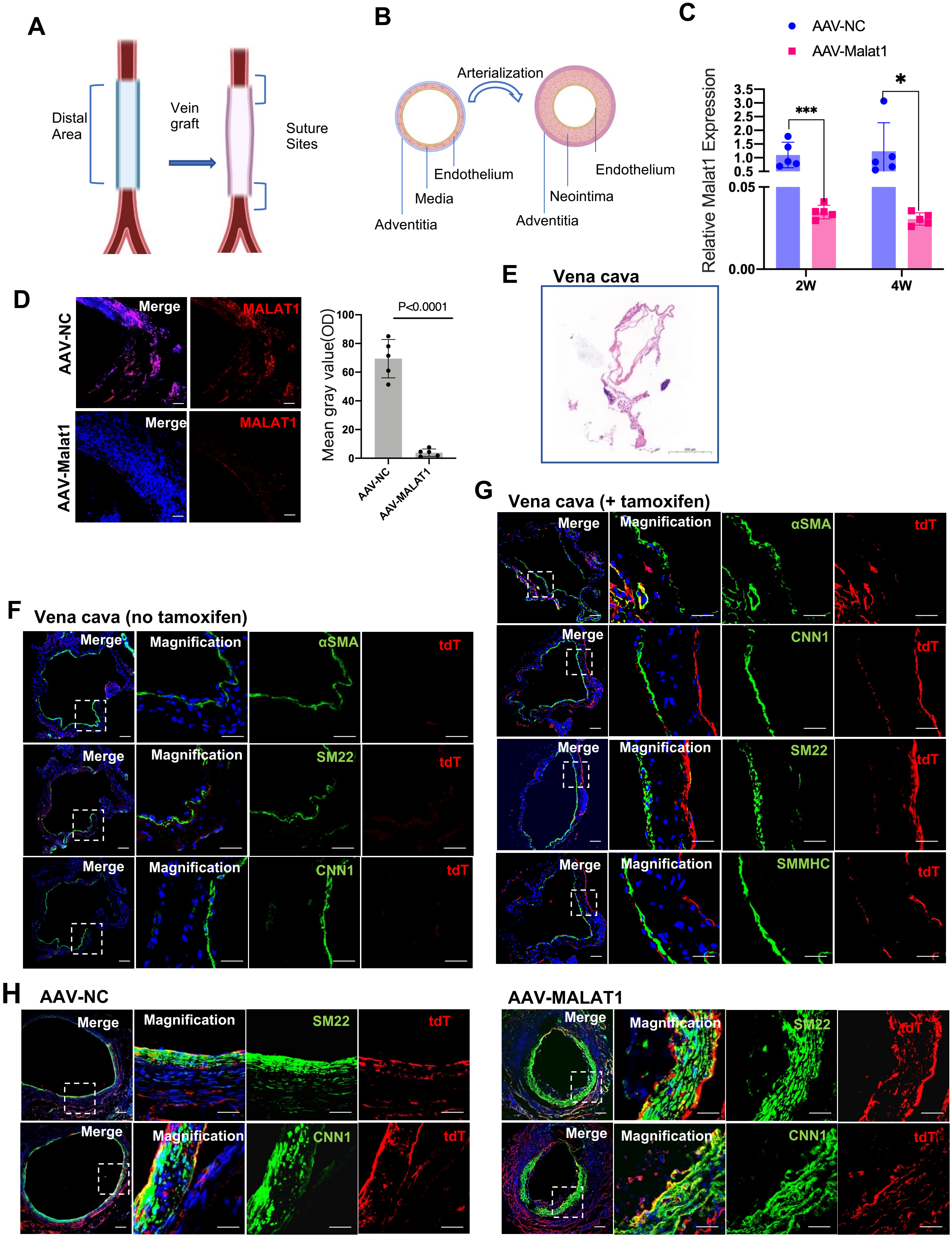
A
Distal Area
Vein graft
Suture Sites
B
Arterialization
Endothelium
Media
Adventitia
Endothelium
Neointima
Adventitia
C
AAV-NC
AAV-Malat1
Relative Malat1 Expression
***
*
2W
4W
D
AAV-NC
AAV-Malat1
Merge
MALAT1
Mean gray value(OD)
P<0.0001
AAV-NC
AAV-MALAT1
E
Vena cava
F
Vena cava (no tamoxifen)
Merge
Magnification
αSMA
SM22
CNN1
tdT
G
Vena cava (+ tamoxifen)
Merge
Magnification
αSMA
CNN1
SM22
SMMHC
tdT
H
AAV-NC
AAV-MALAT1
Merge
Magnification
SM22
CNN1
tdT

Fig. S2 Malat1 deletion promotes SMC generation from Sca1^{+} SPCs in mice vein graft.
A, Schematic representation of the vein graft，showing different zones of the grafted vessel. **B**, A cross-section showing different layers of the vena cava before and after vascular transplantation surgery. **C**, qPCR showing knockdown of Malat1 in veingraft at the second and 4th week with adeno-associated virus. **D**, FISH assay showing the knockdown of Malat1 in veingraft at the 4th week. **E**, HE staining of vena cava before vessel remodeling. Scale bars, 500 μm. **F**, αSMA, SM22 and CNN1 with tdT staining on vena cava from Sca1 lineage tracing mice without tamoxifen treatment. Scale bars, 50 μm. **G**, αSMA, SM22, CNN1 and SMMHC with tdT staining on vena cava from Sca1 lineage tracing mice with tamoxifen treatment. Scale bars, 50 μm. **H**, Fluorescence staining of distal area of veingraft for SM22 and CNN1 with tdTomato in AAV-NC and AAV-Malat1 groups. Scale bars, 100 μm. ***$P < 0.001$, *$P < 0.05$.

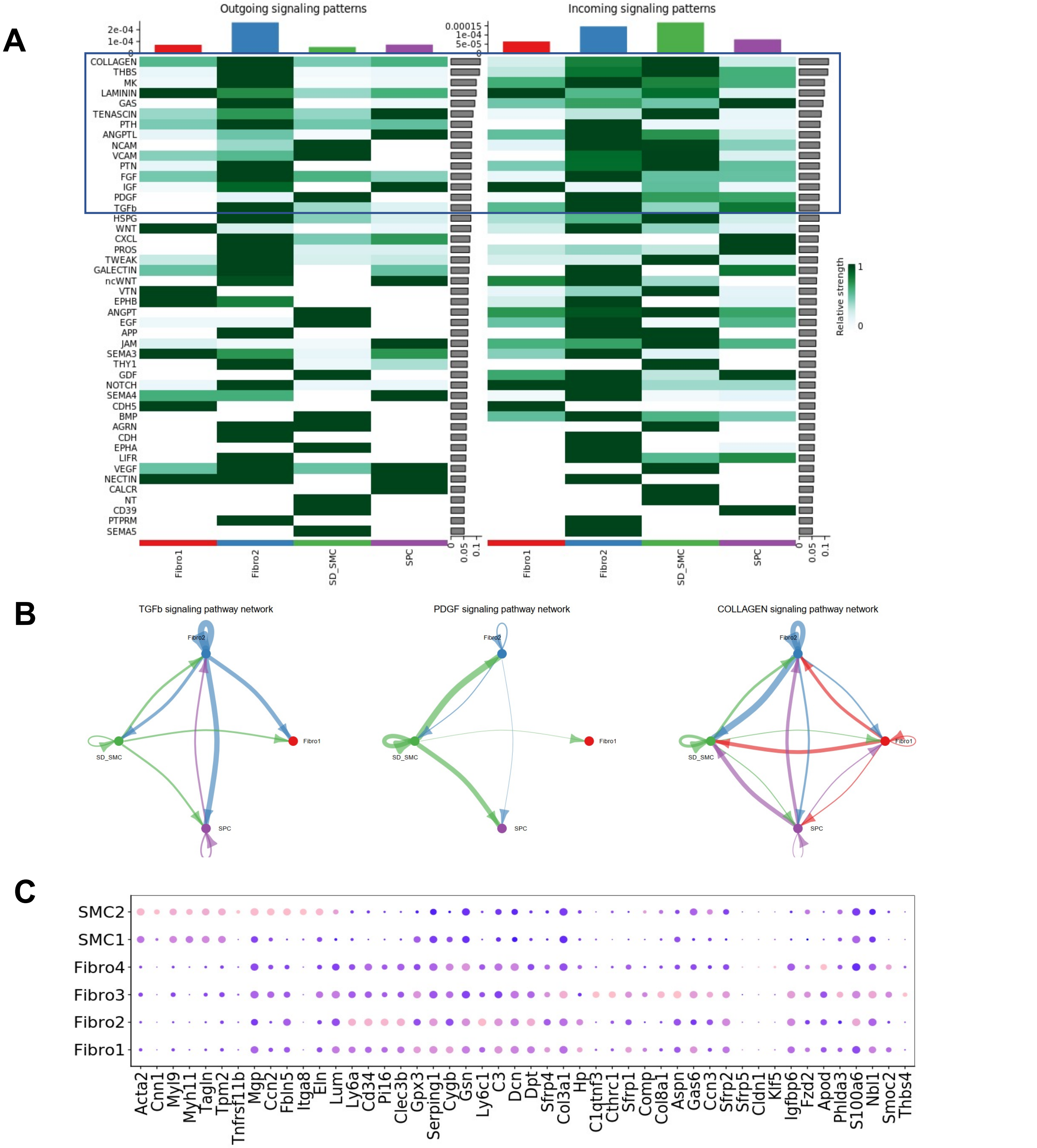

Fig. S3 Intercellular analysis of selected clusters in Fig. 5B and dotplot of Fig. 6B.
A, Heatmap of outgoing and incoming signaling patterns of SPC, SD_SMC, Fibro1 and Fibro2 from Figure 5B. **B**, Circle plot showing TGFβ, PDGF, and Collagen pathways between SPC, SD_SMC, Fibro1, and Fibro2. **C**, A dotplot illustrating the canonical and top expressed genes of each cluster from Fig. 6B.

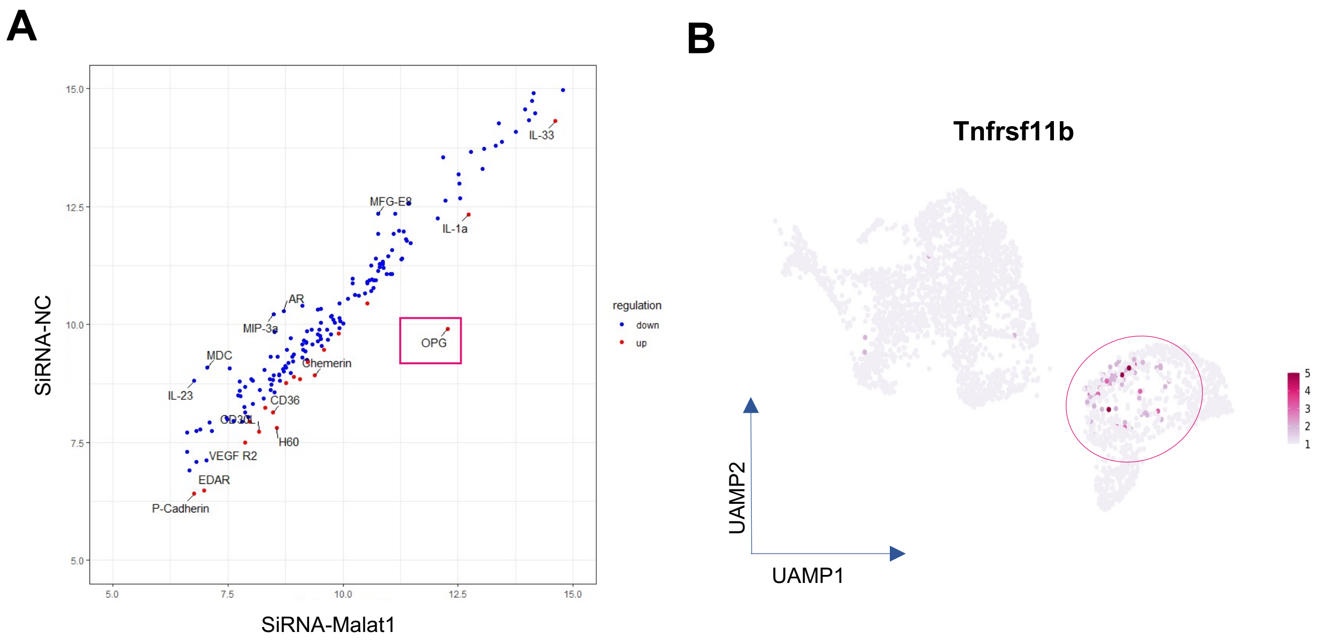

Fig. S4 Protein chip sequencing of Sca1$^+$ cells after knocking down Malat1.
A, Differently expressed proteins in cultured Sca1$^+$ cells after knocking down Malat1, red represents up-regulated and blue represents down-regulated. **B**, Feature plot of Tnfrsf11b (encoding OPG) on the Umap of Figure 5B.

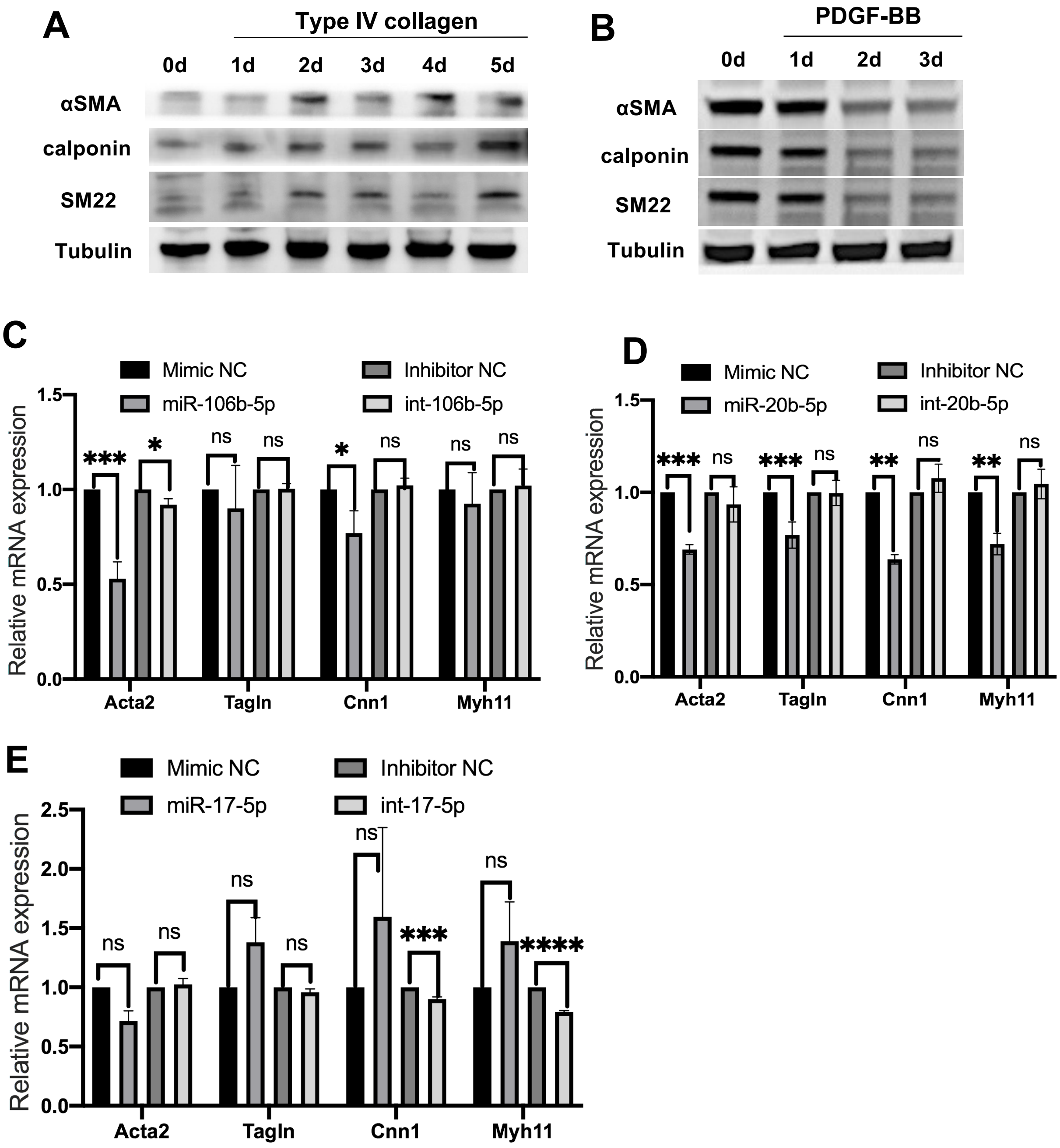

Fig. S5 The effect of Type IV collagen, PDGF-BB and other microRNA candidates on Sca1+ SPCs differentiation into SMCs *in vitro*.

A and **B**，Protein expression level of αSMA, SM22 and calponin as determined by western blotting assay at different time points after culture with Type IV collagen or PDGF-BB. **C-E**, mRNA expression level of Acta2, Tagln, Cnn1, and Myh11 in Sca1+ SPCs transfected with mimic/inhibitor of miR-106b-5p, miR-20b-5p, miR-17-5p and cultured in TGF-β1 as measured using RT-PCR. Data are shown as the Mean ± SD. ****P < 0.0001, ***P < 0.001, ** P < 0.01, *P < 0.05, P ≥ 0.05.
