# Supplementary material for: Fate mapping RNA-sequencing reveal Malat1 regulates Sca1+ progenitor cells to vascular smooth muscle cells transition in vascular remodeling
Source: Cell Mol Life Sci. 2023 Apr 6;80(5):118. doi: 10.1007/s00018-023-04762-3 (PMC10079726; doi:10.1007/s00018-023-04762-3)
Supplement: Supplementary file 2 — Supplementary file2 (DOCX 25 KB) [file 18_2023_4762_MOESM2_ESM.docx]

| Patient information | Vein (n=12) | AVF (n=8) | P-value |
| --- | --- | --- | --- |
| Male sex | 6 (50%) | 7 (87.5%) | 0.085* |
| Age (years) | 62.75 (16.59） | 37.00 (9.35) | 0.091 |
| BMI (kg/m^2^) | 20.79 (2.32) | 22.92 (3.84) | 0.101 |
| Heart rate (bpm) | 76.75 (12.48) | 80.75 (9.84) | 0.783 |
| SBP (mmHg) | 133.33 (18.78) | 137.38 (11.36) | 0.373 |
| DBP (mmHg) | 75.08 (8.50) | 88.25 (16.39) | **0.027** |
| Hypertension | 11 (91.7%) | 5 (62.5%) | 0.110* |
| Diabetes | 3 (25.0%) | 0 (0.0%) | 0.125* |
| Cardiovascular Disease | 3 (25.0%) | 1 (12.5%) | 0.494* |
| CCB | 10 (83.3%) | 5 (62.5%) | 0.539* |
| β blocker | 5 (41.7%) | 4 (50%) | 0.515* |
| ACEI/ARB | 2 (16.7%) | 1 (12.5%) | 0.719* |
| Antiplatelet | 3 (25.0%) | 0 (0.0%) | 0.125* |
| Triglycerides (mM/L) | 1.14 (0.51) | 1.74 (1.15) | 0.069 |
| Total cholesterol (mM/L) | 3.55 (0.71) | 4.95 (0.82) | 0.694 |
| HDL cholesterol (mM/L) | 0.99 (0.37) | 1.28 (0.34) | 0.660 |
| LDL cholesterol (mM/L) | 1.86 (0.62) | 2.92 (0.62) | 0.917 |
| VLDL cholesterol (mM/L) | 0.76 (0.33) | 0.83 (0.39) | 0.881 |
| CKD-EPI(cr) (ml/min) | 8.60 (4.41) | 65.73 (20.80) | **0.014** |
| SCr (μmol/L) | 570.25 (171.40) | 126.25 (39.05) | **0.027** |
| Hemoglobin (g/L) | 94.50 (19.68) | 148.00 (19.96) | 0.627 |
| APTT | 27.48 (3.58) | 26.78 (3.26) | 0.902 |
| PT | 11.53 (0.76) | 10.89 (0.94) | 0.699 |
| D-dimer | 1830.58 (1491.57) | 218.63 (137.53) | **0.003** |

Table S1 Hematological and clinical parameters of patients in this study

Data are n (%) or mean (SD). Statistical analysis, t test, unless otherwise specified. *χ^2^ test. Bold denotes statistical difference. BMI, Body Mass Index, SBP, Systolic Blood Pressure, DBP, Diastolic Blood Pressure, CCB, Calcium Channel Blocker, ACEI/ARB, Angiotensin Converting Enzyme Inhibitors (ACEI)/Angiotensin-Receptor Blockers, CKD-EPI (cr), the serum creatinine-based Chronic Kidney Disease-Epidemiology Collaboration, SCr, Serum Creatinine.
